# Syntheses and structures of a nitro­gen-rich pyrimidine triazole ligand and its Cu^I^ and Ag^I^ complexes

**DOI:** 10.1107/S2056989025009454

**Published:** 2025-10-31

**Authors:** Dylan J. Webb, Elise A. Bennett, René T. Boeré

**Affiliations:** a Department of Chemistry & Physics, Mount Royal University, Calgary, Alberta, Canada, T3E 6K6; b Department of Chemistry and Biochemistry, and, The Canadian Centre for Advanced Fluorine Technologies, University of Lethbridge, Lethbridge, AB, T1K3M4, Canada; University of Aberdeen, United Kingdom

**Keywords:** crystal structure, Cu**^I^** complex, Ag**^I^** complex, pyrimidine, triazole, synthesis

## Abstract

The product of an azide–alkyne Huisgen cyclo­addition between a pyridine azide and a simple alkyne was crystallized alongside the products of the corresponding complexation reactions with copper(I) iodide and silver(I) nitrate. 4,6-Di­meth­yl-2-(4-propyl-1*H*-1,2,3-triazol-1-yl)pyrimidine, C_11_H_15_N_5_, crystallizes with three mol­ecules in the asymmetric unit. The extended structure involves aromatic π-stacking and an extensive network of C—H⋯N hydrogen bonds. The copper complex, di-μ-iodido-bis­{[4,6-dimethyl-2-(4-propyl-1*H*-1,2,3-triazol-1-yl-κ*N*^2^)pyrimidine-κ*N*^1^]copper(I)}, [Cu_2_I_2_(C_11_H_15_N_5_)_2_], is dimeric with a pair of bridging iodide ions and *N*,*N*-chelating ligands, whereas the silver com­plex, bis­[4,6-dimethyl-2-(4-propyl-1*H*-1,2,3-triazol-1-yl-κ*N*^2^)pyrimidine-κ*N*^1^](nitrato-κ*O*)silver(I), [Ag(NO_3_)(C_11_H_15_N_5_)_2_], sees the silver(I) ion suspended between two ligands and weakly attached to a nitrate counter-ion. The copper coordination polyhedra are distorted CuN_2_I_2_ tetra­hedra whereas the silver ion adopts an irregular AgN_4_O coordination polyhedron. The goal of this work is to develop the synthesis of this ligand for the use with natural products as starting materials. The results presented here represent the potential crystallinity of the ligand system and the complexes yielding from it.

## Abstract 2

The product of an azide–alkyne Huisgen cyclo­addition between a pyridine azide and a simple alkyne was crystallized alongside the products of the corresponding complexation reactions with copper(I) iodide and silver(I) nitrate.

## 1. Chemical context

In an attempt to generate a neutral ligand that contains a variety of donating groups suitable to be coordinated by a range of metal centers, a commercially available pyrimidine starting material (4,6-dimethyl-2-(methyl­sulfon­yl)pyrimidine) was used to generate a tetra­zole (3,5-di­methyl­tetra­zolo[1,5*a*]pyrimidine); this tetra­zole was in equilibrium with the organic azide portion (2-azido-4,6-di­methyl­pyrimdine) in solution (Temple & Montgomery, 1964 ▸) making it safer than most organic azides to store (Keicher & Löbbecke, 2009 ▸; Treitler & Leung, 2022 ▸). The energetic functional group was utilized in an azide–alkyne Huisgen cyclo­addition to yield the nitro­gen rich product, 2-(4-propyl-1*H*-1,2,3-triazol-1-yl)-4,6-di­methyl­pyrimidine, C_11_H_15_N_5_, (**I**).

The presence of a pyrimidine moiety in a ligand system provides an effective neutral donor; with the addition of a triazole group, more neutral nitro­gen donors have been introduced creating a malleable environment for coordination (Crowley & McMorran, 2012 ▸; Ségaud *et al.*, 2013 ▸; Štefane *et al.*, 2015 ▸). Both the pyrimidine and the triazole moieties have demonstrated notable medicinal bioactivity (Lagoja, 2005 ▸; Zhou & Wang, 2012 ▸; Sathish Kumar & Kavitha, 2013 ▸) and are therefore of inter­est when designing new compounds, including ligands. There are many pyrimidine analogues, giving a rich and diverse number of compounds including many well-described natural products, including the nucleobases, vitamins, and those derived for pharmaceutical use (Rani *et al.*, 2016 ▸; Kumar *et al.*, 2019 ▸; Nadar & Khan, 2022 ▸). Rather than using these natural products in synthesis, it was of inter­est to use a simple starting material and develop a methodology that could then be adapted to involve a pyrimidine containing natural product as the starting material. Further studies are adapting this procedure with various pyrimidine containing natural products, such as adenine, where the substitution of an aromatic amine is well described (Akhtar *et al.*, 2022 ▸). With the potential of coordination by a metal ion, as seen here with {di-μ-iodo}{[2-(4-propyl-1*H*-1,2,3-triazol-1-yl)-4,6-di­methyl­pyrimidine]-κ^2^-*N*^1^,*N*^4^}copper(I), [Cu_2_I_2_(C_11_H_15_N_5_)_2_], (**II**) and nitrato{*bis*[2-(4-propyl-1*H*-1,2,3-triazol-1-yl)-4,6-di­methyl­pyrimidine]-κ^2^-*N*^1^,*N*^4^}silver(I), [Ag(NO_3_)(C_11_H_15_N_5_)_2_] (**III**), more directed or enhanced bioactivity could be achieved with other green metals—a hitherto unpublished topic.
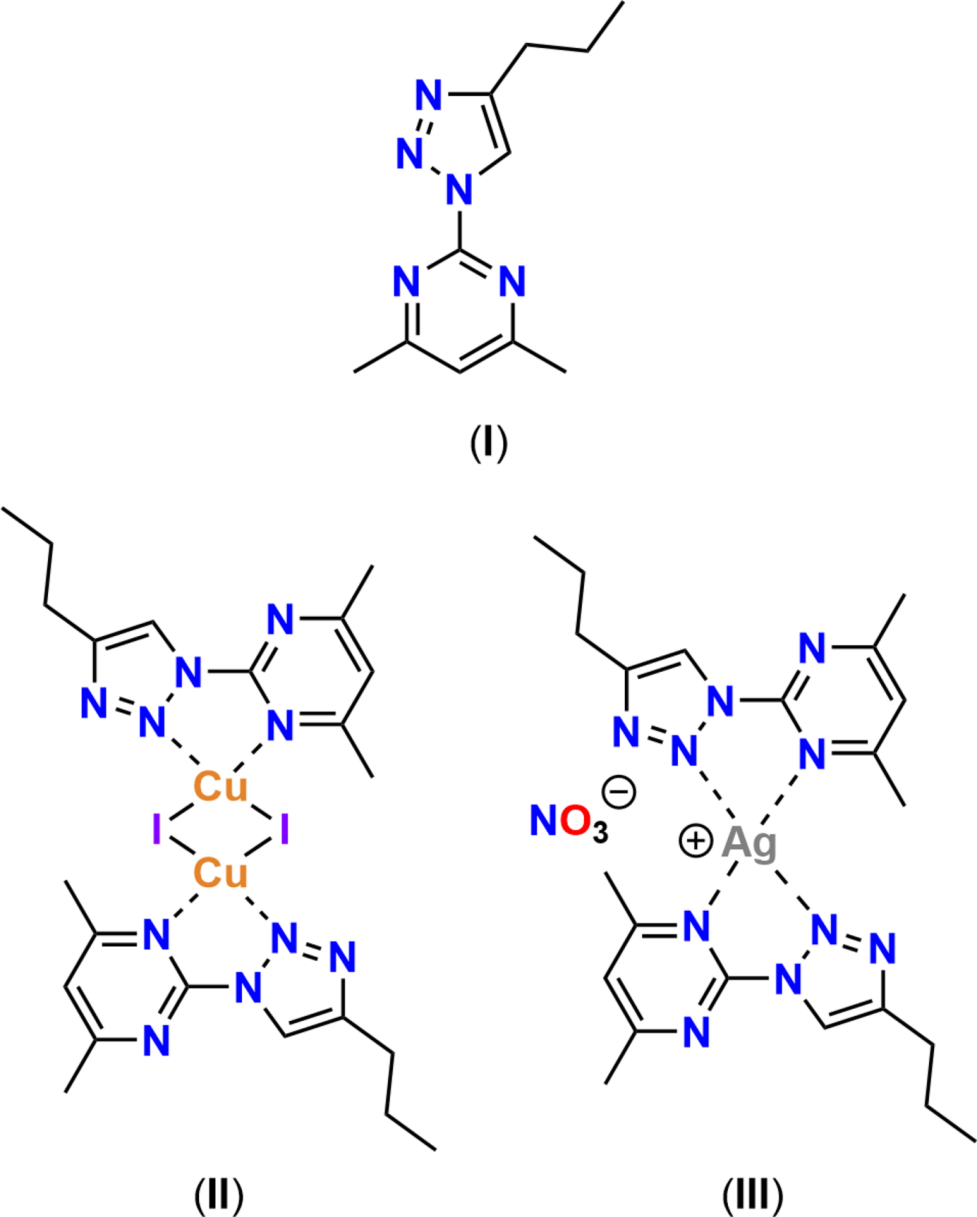


What has installed additional confidence in this ligand system is the crystallinity of the organic fragment, as both the ligand and the Cu^I^ and Ag^I^ complexes readily crystallized, providing high-quality crystals. The crystallinity is theorized to be due to the presence of the triazole group and the propyl chain, but regardless, it bodes well for similar ligands and complexes in the future as they can be further studied and characterized in the solid state.

## 2. Structural commentary

**Figure 1:**
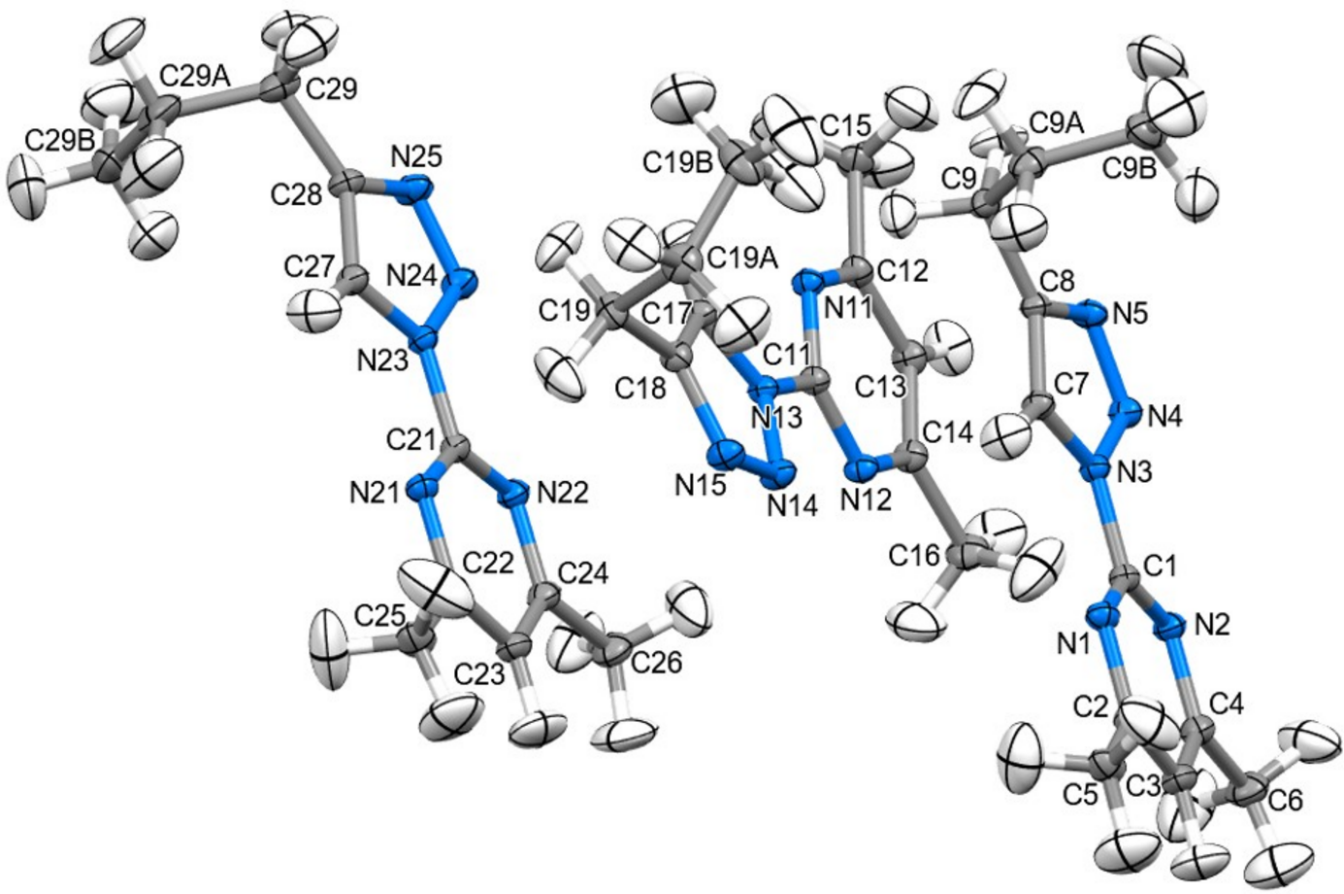
The mol­ecular structure of (**I**) showing 40% displacement ellipsoids for all atoms including H atoms.

The mol­ecular structures of (**I**), (**II**) and (**III**) are shown in Figs. 1 ▸, 2 ▸ and 3 ▸, respectively.

The *Z*′ = 3 structure of (**I**) has three very similar geometries for the 2-(4-propyl-1*H*-1,2,3-triazol-1-yl)-4,6-di­methyl­pyrim­idine heterocycles. The essentially planar triazole (s.d. ≤ 0.002 Å) and pyrimidine (s.d. ≤ 0.005 Å) rings are twisted slightly at the C1{11,21}—N3{13,23} single bonds, with twist angles between the ring planes of 8.96 (3), 11.60 (3) and 8.1 (3)°. All three *n*-propyl groups adopt similar conformations in which the terminal C_2_H_5_ moieties are twisted strongly out of plane. A comparison of the equivalent bonds and angles in each of the unique mol­ecules in the asymmetric unit of (**I**) and the ligands of (**II**) and (**III**) showed little variation with the pyrimidine ring and the triazole ring for both the native ligand and the complexed ligands (summarized in Table 1 ▸). The reported bond lengths and angles for the ring structures do not deviate significantly from the expected data for these functional groups (Constanti­nides *et al.*, 2021 ▸; Amaral *et al.*, 2010 ▸; Rachwal & Katritzky, 2008 ▸): the lack of deviation of the functional groups expressed by the complexes represent little to no disruption of the ligand moiety electronic structure. Longer ligand-to-metal bonds are apparent for (**III**) [2.4444 (8) and 2.4578 (9) Å] compared to (**II**) [2.1274 (9) and 2.0908 (9) Å] whereas the metal-to-counter-ion bonds are longer in (**II**) [2.5985 (1) and 2.5857 (1) Å] compared to (**III**) [2.4035 (11) Å] and similar to the copper to copper distance of 2.5638 (3) Å. Bite angles are consistent for both ligands in either complex, with a smaller angle noted in (**III**) [67.30 (3)°] compared to (**II**) [77.79 (4)°] likely due to the larger Ag^+^ ion.

## 3. Supra­molecular features

The packing of the three similar π-stacked rings [best described by the C18⋯C11 and C18⋯C21 contact distances of 3.2511 (7) and 3.2311 (7) Å, respectively] in the asymmetric unit of (**I**) form layers perpendicular to the *c*-glide planes with a 2_1_ screw axis through the middle layer (Fig. 4 ▸). These mol­ecules additionally inter­act between layers, and laterally within the layers *via* a network of numerous non-classical C—H⋯N hydrogen bonds (Table 2 ▸). The *D*⋯*A* distances range from 3.3359 (8) to 3.6628 (8) Å, and whilst the three shortest contacts have D—H⋯A angles > 160°, are all categorized as ‘weak’ in the classification regime of Jeffrey (Boeré, 2023 ▸). Consistently, the structure possesses a normal density and a mid-range *PLATON* packing index of 0.71 for standard organic crystals.

**Figure 2:**
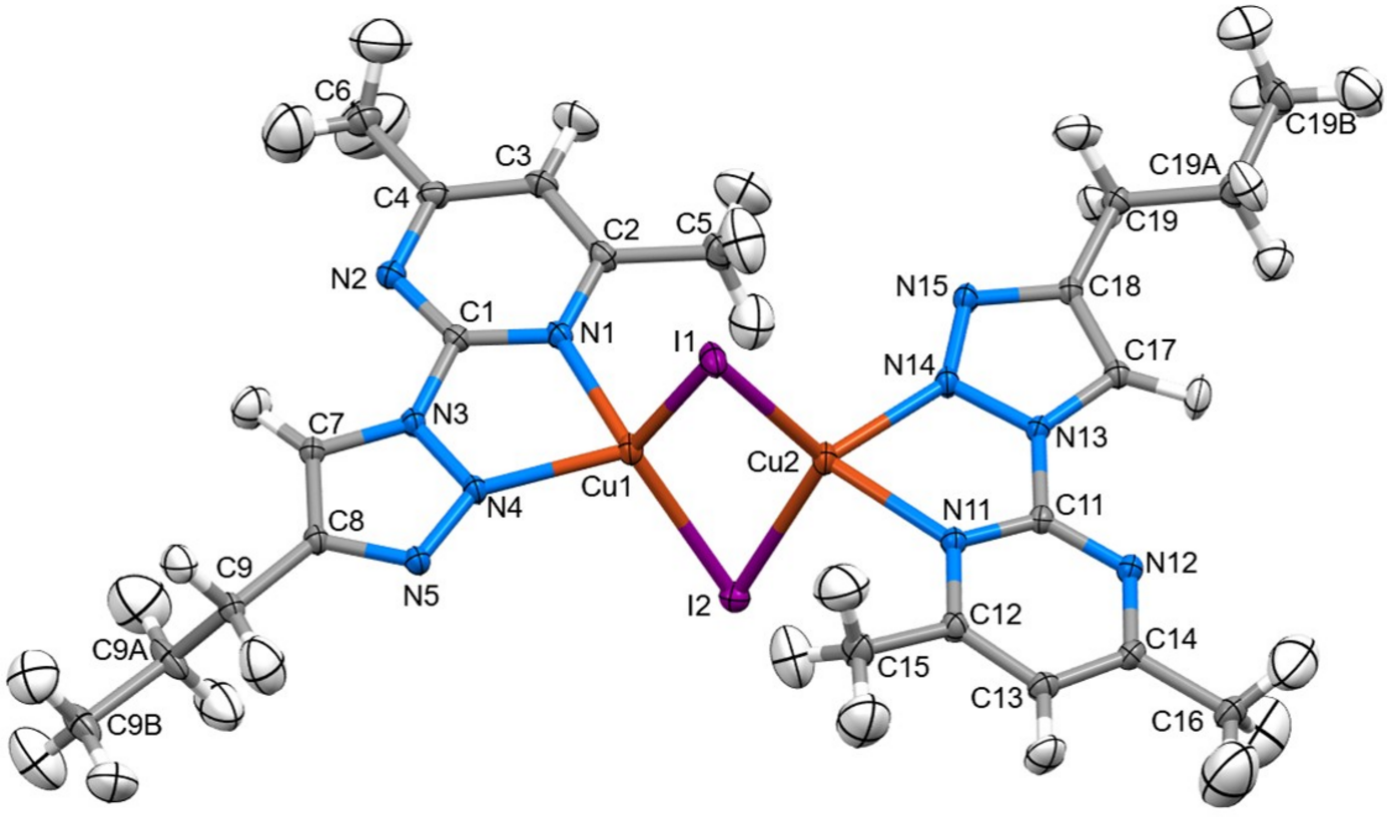
The mol­ecular structure of (**II**) showing 40% displacement ellipsoids for all atoms including H atoms.

**Figure 4:**
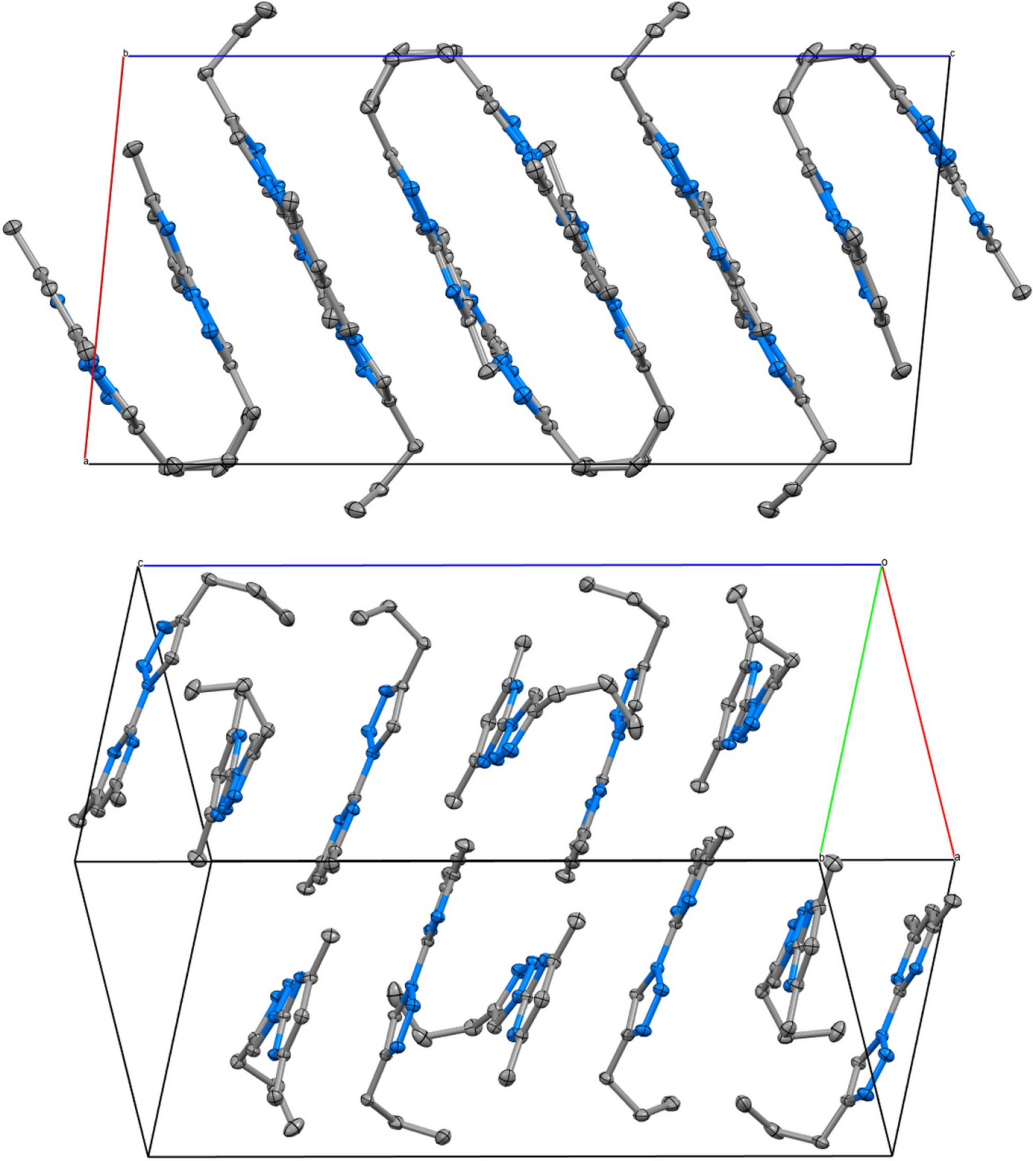
Packing diagrams showing the unit-cell boundaries for two views of (**I**). (Top) a view along the *b-*axis direction, and (bottom) a view that bis­ects the *a* and *b* axes.

**Figure 5:**
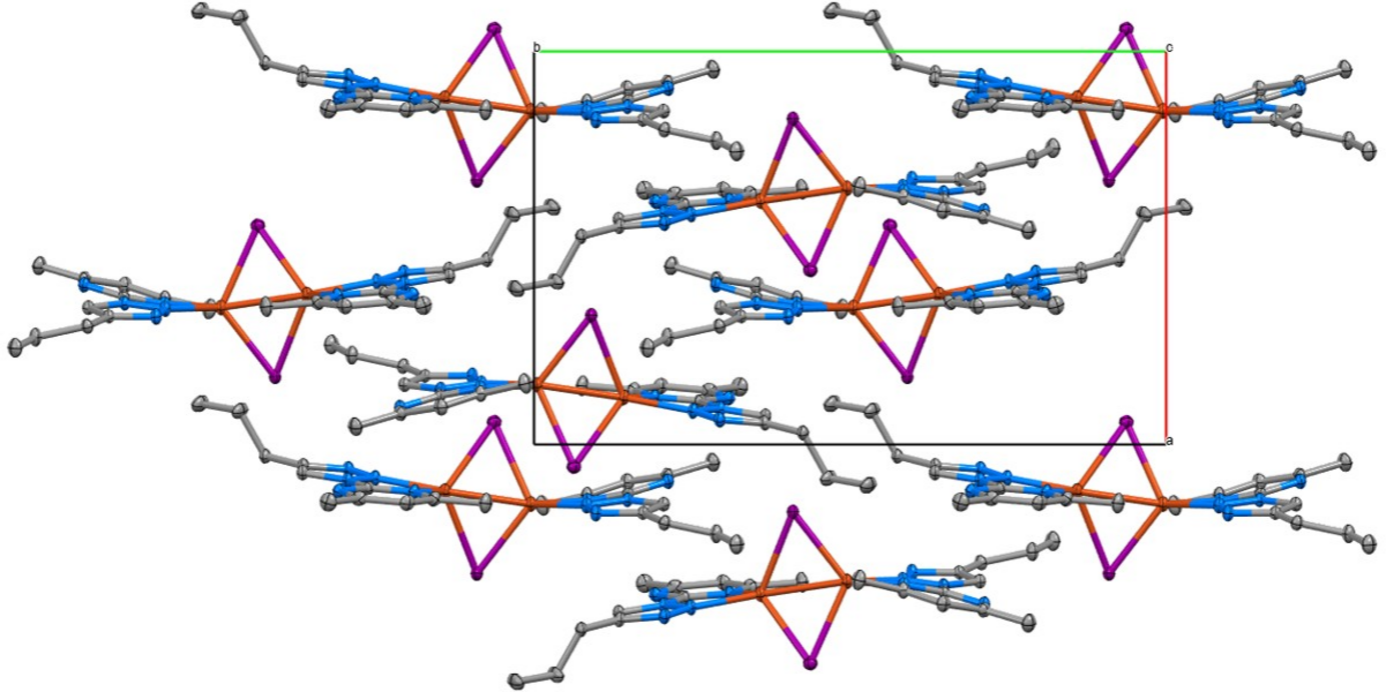
Packing diagram for copper complex (**II**) viewing down the *c* axis showing all mol­ecules that are partly within the unit cell and the bi-directional π-stacking structure that propagates throughout.

The mol­ecular structure of (**II**) whereby the diiodide-bridged Cu**^I^** ions [Cu1⋯Cu2 = 2.5638 (3) Å] are in an almost linear array between two ligands (**I**) that are close to co-planar with each other and with the copper ions (Fig. 2 ▸) is found in the extended structure (Fig. 5 ▸) to form essentially π-stacked layers lying perpendicular to the *a* axis. The shortest contacts from Cu1 to H19*A* (0.43 < ∑*r*_vdW_) are probably incidental to this π-stacking. The next-shortest contacts are non-classical hydrogen bonds (Table 3 ▸) from H17 to I1 (0.42 < ∑*r*_vdW_), which link π-stacked pairs laterally in the *bc* plane as do H16*C* to N5 (0.32 < ∑*r*_vdW_) and H15*C* to I2 (0.22 < ∑*r*_vdW_). A corrugated layer approximately in the *bc* plane develops, from which the bridging iodide ions protrude equally above and below. Trimeric arrays of π-stacking contacts, with C17 to N5 (0.18 < ∑*r*_vdW_) and C17 to C3 (0.12 < ∑*r*_vdW_) develop which step up/down one layer along the corrugated layers with each bridged pair of complexes.

**Figure 6:**
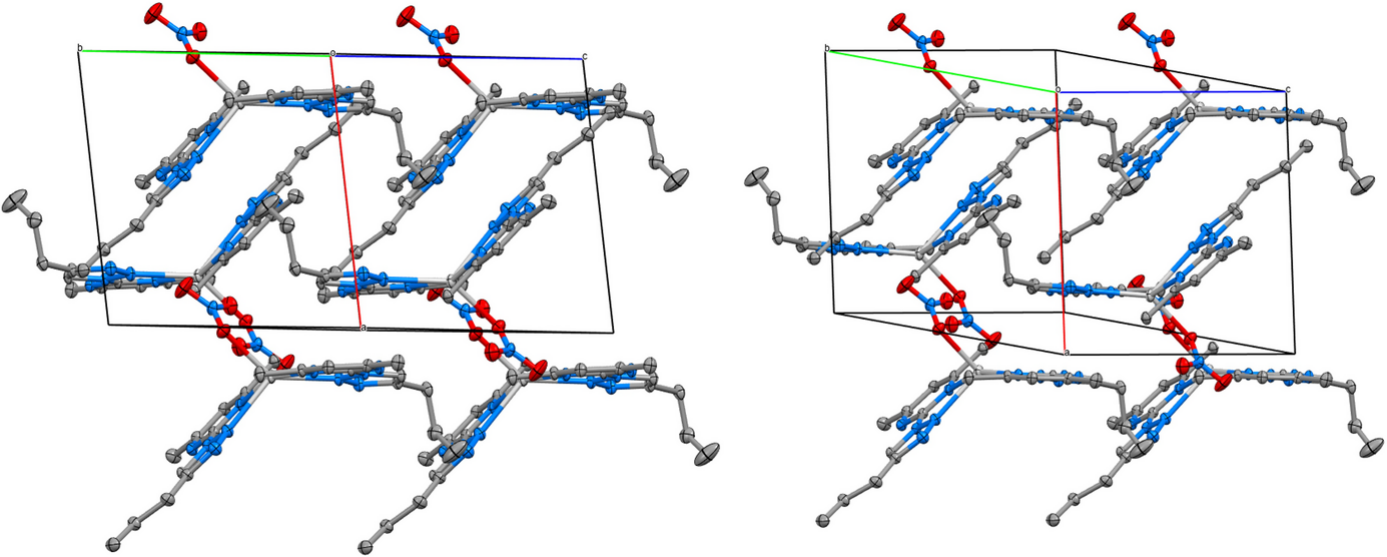
Packing diagram for silver complex (**III**) showing (left) a view through the *a* vertex, bis­ecting the *b* and *c* axes and (right) an alternative view showing the π-stacking structure occurring between ligands of neighbouring complexes.

In the supra­molecular structure of (**III**), similar non-classical hydrogen bonds (Table 4 ▸) are found between aryl- and alkyl C—H bonds, here to both ring N atoms as well as to nitrato O. But the shortest contacts by far involve the large Ag^+^ ion whose coordination is not satisfied by the four ring nitro­gen-atom donors N1, N4, N11 and N14. There are very short contact to the nitrato O atoms, especially O1 [2.404 (1) Å to Ag1], as well as to the C7=C8 double bond of a triazole ring of a neighbouring complex, [Ag⋯C7 = 3.743 (1) Å, some 0.56 Å less than the sum of van der Waals’ radii] (Fig. 6 ▸). Whether a consequence or driving force, this pairing also involves the centrosymmetric π-stacking of pyrazine–triazole rings with a separation of L.S. planes of 3.387 Å, a pattern that is also reminiscent of the free ligands (**I**) and the complex (**II**). A final short contact from Ag^+^ to a ligand methyl C atom occurs in the opposite direction, which is associated with yet another centrosymmetric π-stacked ring to the second ligand rings, this one with a separation of 3.303 Å.

## 4. Database survey

A survey of the Cambridge Structural Database (CSD version 2024.3.1; Groom *et al.*, 2016 ▸) confirmed that the compounds reported here are new. A search with a featureless pyrimidine ring with a nondescript substituted triazole group at the 2 position (2-(4-R-1*H*-1,2,3-triazol-1-yl)pyrimidine), mimicking the core structure of (**I**), revealed eight previously reported structures of organic compounds where none of them contained the same pyrimidine (the 3,5-dimethyl variation) or the same triazole group, the closest being CSD refcode WUYMOU, (ethyl 4-(4-chloro­phen­yl)-6-methyl-2-(4-phenyl-1*H*-1,2,3-triazol-1-yl)pyrimidine-5-carboxyl­ate) (Quan *et al.* 2015 ▸).

In regards to (**I**) acting as a ligand, a search revealed there were three previously reported structures that contained the previously mentioned core structure as a ligand. Of the reported structures, two of them contained single ligands coordinated to a Cu^I^ center, as 1-(pyrimidin-2-yl)-1*H*-benzotriazole, the other with a bridging phosphine ligand, as bis­{μ-[(ethane-1,2-di­yl)bis­(di­phenyl­phosphine)]}-bis­[1-(pyrimidin-2-yl)-1*H*-benzotriazole (Castro *et al.*, 2022 ▸). A similar search where the pyrimidine moiety was replaced with pyridine (one fewer nitro­gen atom in the six-membered aromatic ring) revealed 54 structures. Of these reported structures, two of them involve coordination of the ligand to an Ag^I^ metal center. In addition, both of these Ag^I^ complexes possess quite differing ligand structures, as bis­[μ-2,6-bis­(1,2,3-triazol-1-yl)pyridine]­disilver(I) bis­[μ-2,6-bis­(1,2,3-triazol-1-yl)pyridine]­diaquadi-silver(I) tetra­perchlorate (KANJAO; Capel Berdiell & Halcrow, 2021 ▸) and bis­{μ-[2-{4-[(4-methyl­phen­oxy)meth­yl]-2,3-di­hydro-1*H*-1,2,3-triazol-1-yl}pyridinato]}dinitratodisilver (Gahlaut *et al.* 2023 ▸).

## 5. Synthesis and crystallization

**Figure 7:**
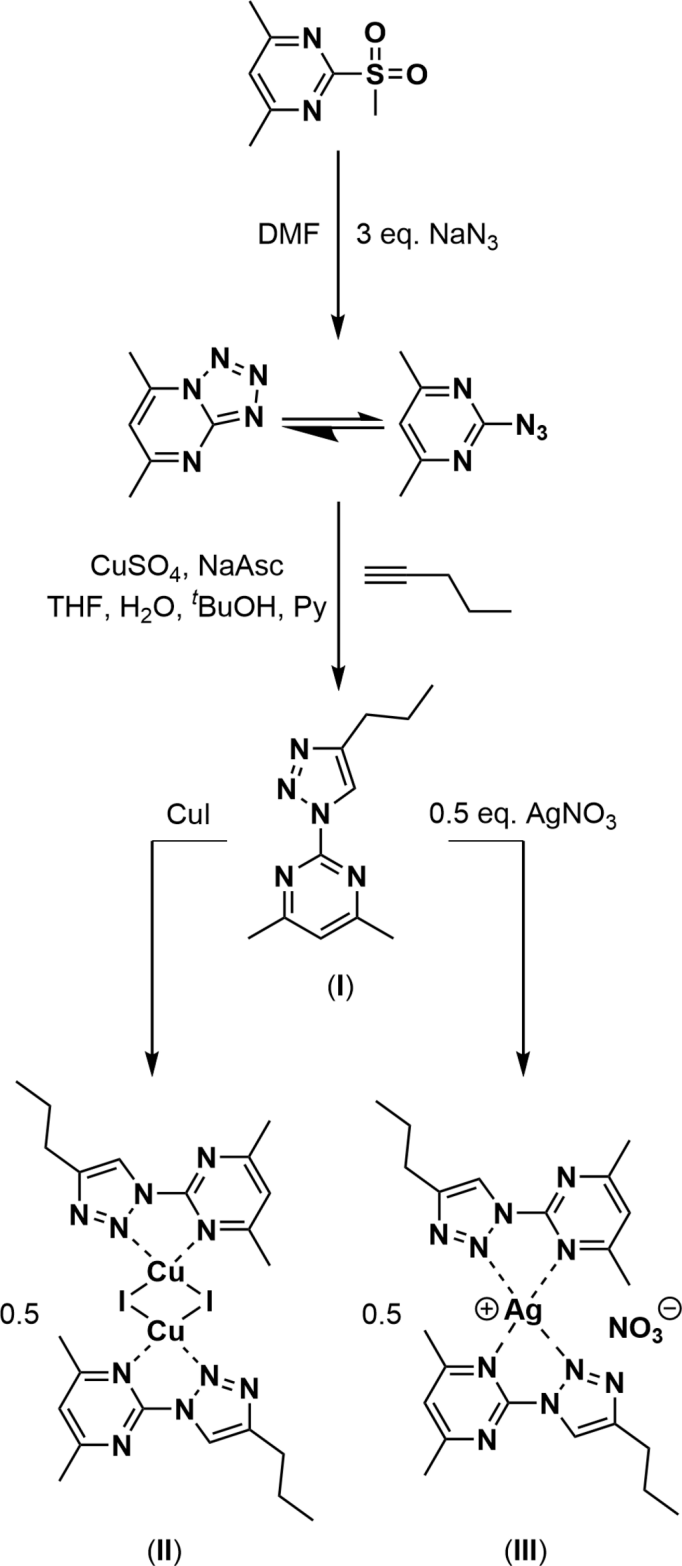
Synthesis scheme of ligand (**I**) and complexes (**II**) and (**III**).

(**I**) A 25 ml Erlenmeyer flask was charged with 4,6-dimethyl-2-(methyl­sulfon­yl)pyrimidine (1.01 g, 5.42 mmol). The flask was then injected with DMF (15 ml) and the resulting citrine-coloured solution stirred. Using a hotplate-mounted sand bath the solution was heated to 373 K to induce a gentle reflux, then left to stir with a Teflon stir bar for 4 h, whereupon no further change was observed. The flask was then allowed to cool to room temperature while continually stirring for 16 h with Parafilm covering the rim of the flask. A yellow–brown turbid layer was apparent in the flask after 16 h. Organics were then transferred to a separatory funnel and rinsed in with 50 ml of dilute salted water. Extraction was then performed with CHCl_3_ washes (∼25 ml × 6), with organic fractions being combined and dried with MgSO_4_. After drying, subsequent solids were removed from the solution *via* gravity filtration to a 250 ml round-bottom flask where bulk solvent was removed *in vacuo* using a rotary evaporator. The residual CHCl_3_ oil was further removed by co-evaporation with hexa­nes before the flask was left under strong vacuum for 48 h. The resulting beige powder consisted of 3,5-di­methyl­tetra­zolo[1,5*a*]pyrimidine. This was used for the subsequent reaction without further purification. The reaction scheme is shown in Fig. 7 ▸.

To the dried tetra­zole was injected a premade solvent system of THF (32 ml), *t*BuOH (32 ml), and distilled water (16 ml). While stirring using a Teflon stir bar, N_2_ gas was bubbled into the solution for 30 min. After bubbling, materials were then added in rapid succession in the sequence: CuSO_4_·5H_2_O (0.39 g, 1.56 mmol); sodium ascorbate (0.73 g, 3.68 mmol); 1-pentyne (0.6 mL, 6.09 mmol); pyridine (3.0 ml, 37.2 mmol). Continuing to stir, N_2_ was bubbled into the flask for an additional 5 min. After which, the flask was plugged by a septum, the septum wrapped in Parafilm, and the entire flask covered in aluminium foil. The solution was then left to stir for 48 h under constant stirring. After 48 h the mixture became neon yellow coloured. The mixture was then suspended in Et_2_O (∼100 ml) and placed in a separatory funnel. Using a premade semi-saturated solution of EDTA in NH_3_ (80 ml), aqueous washes were performed using 10–15 ml of the EDTA solution, at which point the neon yellow color had transitioned to a pale-yellow colour. The organic layer was further washed with a HCl solution (15 ml, 1.0 *M*) followed by brine (50 ml), both of which ran clear. The organic layer was then transferred to a conical flask, dried with MgSO_4_, and subsequent solids removed by gravity filtration. The solution was collected into a 250 ml round-bottom flask and the bulk solvent removed in vacuo using a rotary evaporator, yielding a dark-yellow oil. Residual pyridine persisted following rotary evaporation, it was removed via co-evaporation with hexa­nes, typically using 7–10 hexane rinses (∼2 ml each). Following the hexane washes, the residue was kept under a strong vacuum for 16 h resulting in dark-yellow solid. This solid was found to be the desired product. Yield (0.98 g, 83.1%). ^1^H NMR (90 MHz, chloro­form-*d*_1_) δ 8.35 (*s*, 1H, C^7^), 7.07 (*s*, 1H, C^3^), 2.80 (*t*, ^3^*J*_HH_ = 8.1 Hz, 2H, C^9^), 2.59 (*s*, 6H, C^5^/C^6^), 1.77 (sextet, ^3^*J*_HH_ = 7.2 Hz, 2H, C9*A*), 1.01 (*t*, ^3^*J*_HH_ = 6.3 Hz, 3H, C^9B^). ^13^C{^1^H} NMR (23.6 MHz, chloro­form-*d*_1_) δ 169.6 (*s*, C^2^/C^4^), 154.2 (*s*, C^1^), 148.5 (*s*, C^8^), 119.7 (*s*, C^7^), 119.5 (*s*, C^3^), 27.6 (*s*, C^9^), 24.0 (*s*, C^5^/C^6^), 22.6 (s, C^9A^), 13.7 (*s*, C^9B^).

(**II**) A 25 ml round-bottom flask was charged with (**I**) (0.06 g, 0.27 mmol) and CuI (0.05 g, 0.26 mmol). The flask was injected with acetone (2 ml) and left to stir with a Teflon stir bar until the CuI fully dissolved. The flask was then sealed by a septum and Parafilm before being fully wrapped in tin foil as a protecting measure against photolysis of the forming complex, and left to stir for 30 min, whereupon a tangerine-coloured solution was observed. Acetone was removed *in vacuo* using a rotary evaporator. Remaining solvent was expelled via co-evaporation with hexa­nes, typically 2–3 hexane rinses (∼2 ml each). Following co-evaporation, solids were redissolved in a minimal qu­antity of DMSO, and left in the fume hood for 7 days, wrapped in tinfoil with the mouth of the flask exposed to air. After a week, dark-orange crystals were found adhered to the flask and the remaining clear DMSO solution was poured off the crystals. Crystals were dried with one additional hexane rinse (2 ml) and left under high vac for 1 h. The resulting dark-orange crystals were found to be the desired product, which is stable under ambient light and air. Yield 0.09 g, 81.8%. ^1^H NMR (90 MHz, chloro­form-*d*_1_) δ 8.39 (*s*, 1H, C^7^), 7.16 (*s*, 1H, C^3^), 2.94 (*m*, 2H, C^9^), 2.73 (*s*, 6H, C^5^/C^6^), 1.83 (sextet, ^3^*J*_HH_ = 7.2 Hz, 2H, C^9*A*^), 1.04 (*t*, ^3^*J*_HH_ = 7.2 Hz, 3H, C^9*B*^). ^13^C{^1^H} NMR (23.6 MHz, chloro­form-*d*_1_) δ 169.6 (*s*, C^2^/C^4^), 153.7 (*s*, C^1^), 148.9 (*s*, C^8^), 119.7 (*s*, C^7^), 112.6 (*s*, C^3^), 27.7 (*s*, C^9^), 24.2 (*s*, C^5^/C^6^), 22.5 (*s*, C^9*A*^), 13.8 (*s*, C^9*B*^).

(**III**) A 25 ml round-bottom flask was charged with (**I**) (0.14 g, 0.64 mmol) and AgNO_3_ (0.05 g, 0.29 mmol). The flask was then injected with EtOH (2 ml) and left to stir with a Teflon stir bar until the AgNO_3_ fully dissolved. The flask was then sealed by a septum and Parafilm before being fully wrapped in tin foil, as a protecting measure against photolysis of the forming complex, and left to stir for 72 h, whereupon the still clear solvent was observed with white–beige solids deposited on the flask. EtOH was removed *in vacuo* using a rotary evaporator. Excess free ligand was triturated out of the flask via chloro­form rinses, and remaining solvent expelled *via* co-evaporation with hexa­nes, typically 3–5 hexane rinses (∼2 ml each). Following co-evaporation, the solids were left under a strong vacuum for an additional 16 h, resulting in a refined beige powder, which was found to be the desired product. Yield 0.09 g (60.0%) ^1^H NMR (90 MHz, DMSO-*d*_6_) δ 8.67 (*s*, 1H, C^7^), 7.45 (*s*, 1H, C^3^), 2.71 (*t*, ^3^*J*_HH_ = 7.2 Hz, 2H, C^9^), 2.53 (*s*, 6H, C^5^/C^6^), 1.68 (sextet, ^3^*J*_HH_ = 7.2 Hz, 2H, C^9*A*^), 0.91 (*t*, ^3^*J*_HH_ = 7.2 Hz, 3H, C^9*B*^). ^13^C{^1^H} NMR (23.6 MHz, chloro­form-*d*_1)_ δ 169.5 (*s*, C^2^/C^4^), 154.8 (*s*, C^1^), 148.3 (*s*, C^8^), 120.9 (*s*, C^7^), 120.3 (*s*, C^3^), 26.8 (*s*, C^9^), 23.6 (*s*, C^5^/C^6^), 21.9 (*s*, C^9*A*^), 13.4 (s, C^9*B*^).

For (**I**), crystals suitable for diffraction came from a solution in CHCl_3_ kept in a fridge (277 K) where crystals grew over 2 days. For (**II**) and (**III**), the compounds were dissolved in minimal DMSO and crystals grew over the next week at room temperature.

## 6. Refinement

Crystal data, data collection and structure refinement details are summarized in Table 5 ▸. Refinement of (**I**) was undertaken in *OLEX2* (Bourhis *et al.*, 2015 ▸) using *olex2.refine* and followed by Hirshfeld atom refinement using *NoSpherA2* (Kleemiss *et al.*, 2021 ▸). The ED was calculated from a Gaussian basis set single determinant SCF wavefunction using *ORCA 5.0* at the def2-TZVPP/R2SCAN level of theory. The aspherical atomic scattering factors were calculated by *NoSpherA2* and fed back into *olex2.refine*. In the final cycles of HAR, the ED calculation was repeated 10 times using normal integration accuracy and a solvation model for water to improve the definition of the ED. The LS refinement of the crystal model converged and yielded an average s.u. for the (predominant) C—C bonds of 0.0009 Å that was 44% smaller than the model obtained without HAR with *NoSpherA2*. The refinement of (**II**) followed a similar procedure except that a ZORA-corrected relatavistic x2c-TZVP/R2SCAN level of theory was employed (principally to deal with the iodine atoms). The final average s.u. for C—C bonds of 0.0023 Å that was 23% smaller than the model obtained in the independent atom model. ISOR restraints were applied to the anisotropic displacement refinements of the H atoms. In the refinement of (**III**) a DKH2-corrected relatavistic x2c-TZVP/R2SCAN level of theory was employed. The final average s.u. for C—C bonds was also 0.0023 Å but here is 26% lower than the IAM alternative. For this model, the H-atom refinement required ISOR restraints, and, for the terminal CH_3_ groups of the propyl chains (C9, C19) also DFIX restraints of the C—H distances to the best neutron diffraction estimates in the same temperature range (Allen & Bruno, 2010 ▸). The improvements in s.u. in these three structures may be compared with those reported in a recent review (Hill & Boeré, 2025 ▸).

## Supplementary Material

Crystal structure: contains datablock(s) I, II, III. DOI: 10.1107/S2056989025009454/hb8166sup1.cif

Structure factors: contains datablock(s) I. DOI: 10.1107/S2056989025009454/hb8166Isup2.hkl

Structure factors: contains datablock(s) II. DOI: 10.1107/S2056989025009454/hb8166IIsup3.hkl

Structure factors: contains datablock(s) III. DOI: 10.1107/S2056989025009454/hb8166IIIsup4.hkl

CCDC references: 2498503, 2498504, 2498505

Additional supporting information:  crystallographic information; 3D view; checkCIF report

## Figures and Tables

**Figure 3:**
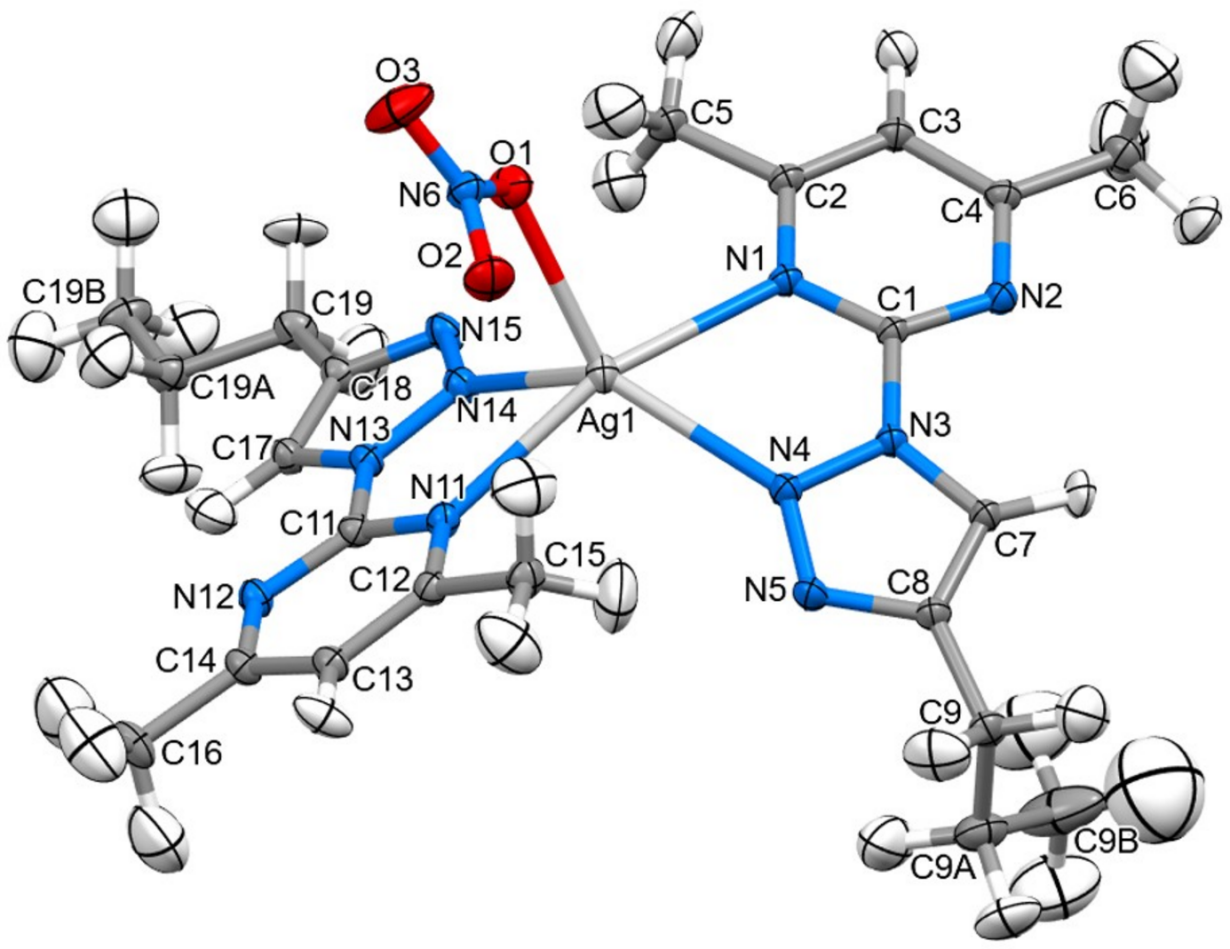
The mol­ecular structure of (**III**) showing 40% displacement ellipsoids for all atoms including H atoms.

**Table 1:** Comparative inter­atomic dimensions (Å, °) The three mol­ecules in the asymmetric unit of (**I**) are summarized together; the two distinct ligands of (**II**) and (**III**) are summarized together. Summarizing was conducted based on the atom-numbering scheme, which was done in parallel for 1, 2 or 3 identical ligand mol­ecules. ‘Ion’ for (**II**) is an iodide ion and for (**III**) is an O atom of a nitrate ion.

Averaged bond lengths	(**I**)	(**II**)	(**III**)	Averaged bond angles	(**I**)	(**II**)	(**III**)
Pyrimidine ring				Pyrimidine ring			
C1—N1	1.3263 (4)	1.3313 (13)	1.3243 (13)	C1—N1—C2	115.44 (3)	115.99 (10)	115.67 (8)
N1—C2	1.3447 (4)	1.3501 (13)	1.3512 (13)	N1—C2—C3	120.76 (3)	119.98 (10)	120.45 (9)
C2—C3	1.3940 (5)	1.3940 (14)	1.3922 (13)	C2—C3—C4	118.21 (3)	118.75 (11)	118.56 (10)
C3—C4	1.3914 (5)	1.3985 (14)	1.3940 (14)	C3—C4—N2	121.25 (3)	120.80 (11)	120.93 (9)
C4—N2	1.3463 (4)	1.3480 (14)	1.3448 (13)	C4—N2—C1	114.98 (3)	115.41 (10)	115.39 (9)
N2—C1	1.3241 (4)	1.3184 (13)	1.3272 (12)	N2—C1—N1	129.34 (3)	129.07 (10)	129.01 (9)
Triazole ring				Triazole ring			
N3—N4	1.3473 (3)	1.3525 (12)	1.3521 (11)	N3—N4—N5	107.18 (2)	107.75 (9)	107.51 (8)
N4—N5	1.3048 (4)	1.3012 (12)	1.3022 (12)	N4—N5—C8	109.75 (2)	109.21 (9)	109.55 (8)
N5—C8	1.3685 (4)	1.3703 (13)	1.3710 (13)	N5—C8—C7	107.63 (3)	108.22 (10)	107.87 (9)
C8—C7	1.3736 (5)	1.3775 (14)	1.3750 (14)	C8—C7—N3	104.75 (3)	104.34 (10)	104.67 (8)
C7—N3	1.3600 (4)	1.3623 (13	1.3635 (13)	C7—N3—N4	110.68 (2)	110.49 (9)	110.41 (8)
Metal–ligand				Metal–ligand			
*M*—N1	–	2.1274 (9)	2.4444 (8)	N1—*M*—N4	–	77.79 (4)	67.30 (3)
*M*—N4	–	2.0908 (9)	2.4578 (9)	N1—*M*—N4′	–	–	103.01 (3)
*M*1—ion	–	2.5985 (1)	2.4035 (11)	*M*—ion—*M*	–	59.27 (5)	–
*M*2—ion	–	2.5857 (1)	–	N1—*M*—*M*	–	136.49 (3)	–
*M*—*M*	–	2.5638 (3)	–	N4—*M*—*M*	–	114.65 (3)	–

**Table 2:** Hydrogen-bond geometry (Å, °) for (**I**)

*D*—H⋯*A*	*D*—H	H⋯*A*	*D*⋯*A*	*D*—H⋯*A*
C3—H3⋯N24^i^	1.074 (7)	2.279 (7)	3.3359 (8)	167.3 (5)
C3—H3⋯N25^i^	1.074 (7)	2.519 (7)	3.5487 (8)	160.2 (5)
C5—H5*a*⋯N25^ii^	1.075 (7)	2.641 (7)	3.6399 (8)	154.2 (6)
C13—H13⋯N14^iii^	1.074 (6)	2.361 (7)	3.4097 (8)	165.1 (5)
C15—H15*c*⋯N12^iii^	1.083 (7)	2.634 (8)	3.6588 (8)	157.7 (5)
C23—H23⋯N4^i^	1.082 (7)	2.300 (7)	3.3753 (8)	172.2 (6)
C23—H23⋯N5^i^	1.082 (7)	2.629 (7)	3.6525 (7)	157.6 (5)
C25—H25*b*⋯N5^iv^	1.083 (8)	2.520 (8)	3.4538 (9)	143.8 (5)

Symmetry codes: (i) 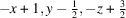; (ii) 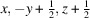; (iii) 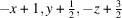; (iv) 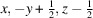.

**Table 3:** Hydrogen-bond geometry (Å, °) for (**II**)

*D*—H⋯*A*	*D*—H	H⋯*A*	*D*⋯*A*	*D*—H⋯*A*
C15—H15C⋯I2^i^	1.06 (2)	3.02 (2)	4.0596 (19)	165 (2)
C16—H16C⋯N5^i^	1.03 (3)	2.54 (3)	3.492 (2)	155 (3)
C17—H17⋯I1^ii^	1.09 (2)	2.82 (2)	3.9016 (16)	171 (2)

Symmetry codes: (i) 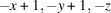; (ii) 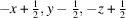.

**Table 4:** Hydrogen-bond geometry (Å, °) for (**III**)

*D*—H⋯*A*	*D*—H	H⋯*A*	*D*⋯*A*	*D*—H⋯*A*
C5—H5A⋯N15	1.07 (3)	2.57 (2)	3.516 (2)	146 (2)
C5—H5B⋯O1	1.11 (2)	2.34 (2)	3.358 (2)	152 (2)
C15—H15A⋯N5	1.08 (2)	2.46 (3)	3.517 (2)	167 (2)
C15—H15B⋯O2	1.09 (2)	2.36 (2)	3.333 (2)	148 (2)
C3—H3⋯O3^i^	1.09 (2)	2.31 (2)	3.378 (2)	167.6 (14)
C7—H7⋯O2^ii^	1.075 (18)	2.369 (18)	3.3044 (19)	144.6 (14)
C9—H9A⋯O1^ii^	1.10 (2)	2.39 (2)	3.449 (2)	162 (2)
C13—H13⋯O2^iii^	1.07 (2)	2.41 (2)	3.391 (2)	152.2 (15)
C17—H17⋯N5^iv^	1.04 (2)	2.53 (2)	3.524 (2)	160.0 (17)
C19—H19B⋯O3^v^	1.09 (2)	2.30 (2)	3.324 (2)	157 (2)

Symmetry codes: (i) 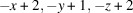; (ii) 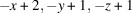; (iii) 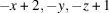; (iv) 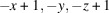; (v) 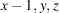.

**Table 5:** Experimental details

	(**I**)	(**II**)	(**III**)
Crystal data			
Chemical formula	C_11_H_15_N_5_	[Cu_2_I_2_(C_11_H_15_N_5_)_2_]	[Ag(NO_3_)(C_11_H_15_N_5_)_2_]
*M*_r_	217.28	815.45	604.42
Crystal system, space group	Monoclinic, *P*2_1_/*c*	Monoclinic, *P*2_1_/*n*	Triclinic, *P* 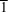
Temperature (K)	100	100	100
*a*, *b*, *c* (Å)	10.99556 (14), 14.2664 (2), 22.1652 (3)	11.5497 (3), 18.2906 (3), 13.7287 (3)	10.4356 (2), 11.4639 (2), 11.8944 (2)
α, β, γ (°)	90, 95.5049 (12), 90	90, 99.489 (2), 90	97.213 (1), 100.878 (2), 110.519 (2)
*V* (Å^3^)	3460.94 (8)	2860.54 (10)	1279.99 (5)
*Z*	12	4	2
Radiation type	Cu *K*α	Mo *K*α	Cu *K*α
μ (mm^−1^)	0.65	3.68	6.72
Crystal size (mm)	0.14 × 0.08 × 0.03	0.23 × 0.11 × 0.09	0.35 × 0.1 × 0.06 × 0.07 (radius)

Data collection			
Diffractometer	SuperNova, Dual, Cu at home/near, Pilatus 200K	SuperNova, Dual, Cu at home/near, Pilatus 200K	SuperNova, Dual, Cu at home/near, Pilatus 200K
Absorption correction	Multi-scan (*CrysAlis PRO*; Rigaku OD, 2024 ▸)	Multi-scan (*CrysAlis PRO*; Rigaku OD, 2024 ▸	For a sphere (*CrysAlis PRO*; Rigaku OD, 2024 ▸)
*T*_min_, *T*_max_	0.923, 1.000	0.745, 1.000	0.369, 0.429
No. of measured, independent and observed [*I* ≥ 2u(*I*)] reflections	34796, 7021, 6059	142732, 8347, 7323	24918, 5150, 4952
*R*_int_	0.028	0.060	0.045
(sin θ/λ)_max_ (Å^−1^)	0.627	0.703	0.626

Refinement			
*R*[*F*^2^ > 2σ(*F*^2^)], *wR*(*F*^2^), *S*	0.019, 0.035, 1.07	0.019, 0.037, 1.02	0.019, 0.046, 1.09
No. of reflections	7021	8347	5150
No. of parameters	838	595	604
No. of restraints	6	189	213
H-atom treatment	All H-atom parameters refined	All H-atom parameters refined	All H-atom parameters refined
Δρ_max_, Δρ_min_ (e Å^−3^)	0.16, −0.13	0.84, −0.64	0.44, −0.53

Computer programs: *CrysAlis PRO* (Rigaku OD, 2024 ▸), *SHELXT* (Sheldrick, 2015 ▸), *OLEX2.refine* (Bourhis *et al.*, 2015 ▸) and *OLEX2* (Dolomanov *et al.*, 2009 ▸).

## supplementary crystallographic information

### 4,6-Dimethyl-2-(4-propyl-1H-1,2,3-triazol-1-yl)pyrimidine (I) Crystal data

**Table:**

C_11_H_15_N_5_	*F*(000) = 1396.488
*M**_r_* = 217.28	*D*_x_ = 1.251 Mg m^−^^3^
Monoclinic, *P*2_1_/*c*	Cu *K*α radiation, λ = 1.54184 Å
*a* = 10.99556 (14) Å	Cell parameters from 16391 reflections
*b* = 14.2664 (2) Å	θ = 3.7–74.8°
*c* = 22.1652 (3) Å	µ = 0.65 mm^−^^1^
β = 95.5049 (12)°	*T* = 100 K
*V* = 3460.94 (8) Å^3^	Trapezoid, clear colourless
*Z* = 12	0.14 × 0.08 × 0.03 mm

### 4,6-Dimethyl-2-(4-propyl-1H-1,2,3-triazol-1-yl)pyrimidine (I) Data collection

**Table:**

SuperNova, Dual, Cu at home/near, Pilatus 200K diffractometer	7021 independent reflections
Radiation source: micro-focus sealed X-ray tube, SuperNova (Cu) X-ray Source	6059 reflections with *I*≥ 2u(*I*)
Mirror monochromator	*R*_int_ = 0.028
Detector resolution: 5.8140 pixels mm^-1^	θ_max_ = 75.0°, θ_min_ = 3.7°
ω scans	*h* = −13→13
Absorption correction: multi-scan (CrysAlisPro; Rigaku OD, 2024)	*k* = −17→13
*T*_min_ = 0.923, *T*_max_ = 1.000	*l* = −27→27
34796 measured reflections	

### 4,6-Dimethyl-2-(4-propyl-1H-1,2,3-triazol-1-yl)pyrimidine (I) Refinement

**Table:**

Refinement on *F*^2^	0 constraints
Least-squares matrix: full	Primary atom site location: dual
*R*[*F*^2^ > 2σ(*F*^2^)] = 0.019	All H-atom parameters refined
*wR*(*F*^2^) = 0.035	*w* = 1/[σ^2^(*F*_o_^2^) + (0.0046*P*)^2^ + 0.0799*P*] where *P* = (*F*_o_^2^ + 2*F*_c_^2^)/3
*S* = 1.07	(Δ/σ)_max_ = 0.001
7021 reflections	Δρ_max_ = 0.16 e Å^−^^3^
838 parameters	Δρ_min_ = −0.13 e Å^−^^3^
6 restraints	

### 4,6-Dimethyl-2-(4-propyl-1H-1,2,3-triazol-1-yl)pyrimidine (I) Fractional atomic coordinates and isotropic or equivalent isotropic displacement parameters (Å^2^)

**Table:**

	*x*	*y*	*z*	*U*_iso_*/*U*_eq_	
N1	0.42083 (4)	0.15598 (3)	0.89170 (2)	0.01652 (10)	
N2	0.57673 (4)	0.26602 (3)	0.92631 (2)	0.01711 (10)	
N3	0.39036 (4)	0.31471 (3)	0.87684 (2)	0.01419 (10)	
N4	0.41627 (4)	0.40545 (3)	0.88897 (2)	0.01666 (10)	
N5	0.32556 (4)	0.45483 (3)	0.86347 (2)	0.01705 (10)	
C1	0.46843 (5)	0.24098 (4)	0.89992 (2)	0.01469 (11)	
C2	0.49219 (5)	0.08482 (4)	0.91344 (2)	0.01771 (12)	
C3	0.60800 (5)	0.10215 (4)	0.94274 (3)	0.02071 (12)	
H3	0.6663 (6)	0.0457 (5)	0.9597 (3)	0.0404 (19)	
C4	0.64756 (5)	0.19462 (4)	0.94839 (3)	0.01923 (12)	
C5	0.44247 (6)	−0.01233 (4)	0.90479 (3)	0.02253 (13)	
H5a	0.3614 (7)	−0.0204 (5)	0.9278 (4)	0.054 (2)	
H5b	0.4173 (7)	−0.0254 (5)	0.8567 (4)	0.053 (2)	
H5c	0.5087 (7)	−0.0649 (5)	0.9219 (4)	0.051 (2)	
C6	0.77198 (6)	0.21948 (6)	0.97792 (4)	0.02781 (14)	
H6a	0.8310 (7)	0.2425 (7)	0.9439 (4)	0.066 (3)	
H6b	0.7678 (7)	0.2777 (6)	1.0094 (4)	0.055 (2)	
H6c	0.8154 (6)	0.1605 (6)	1.0005 (4)	0.055 (2)	
C7	0.28107 (5)	0.30609 (4)	0.84287 (2)	0.01544 (11)	
H7	0.2459 (6)	0.2384 (5)	0.8279 (3)	0.0323 (18)	
C8	0.24004 (5)	0.39656 (4)	0.83449 (2)	0.01486 (11)	
C9	0.12495 (5)	0.43277 (5)	0.80120 (3)	0.01838 (12)	
H9a	0.1261 (6)	0.5106 (5)	0.8040 (3)	0.0344 (18)	
H9b	0.1247 (6)	0.4161 (5)	0.7532 (3)	0.0370 (19)	
C9A	0.00938 (5)	0.39442 (5)	0.82548 (3)	0.02305 (13)	
H9Aa	−0.0700 (6)	0.4160 (5)	0.7943 (3)	0.046 (2)	
H9Ab	0.0121 (6)	0.3166 (5)	0.8246 (3)	0.0400 (19)	
C9B	−0.00702 (7)	0.42725 (6)	0.88950 (3)	0.03045 (15)	
H9Ba	−0.0183 (8)	0.5038 (6)	0.8906 (4)	0.070 (3)	
H9Bb	−0.0880 (7)	0.3953 (6)	0.9063 (4)	0.061 (3)	
H9Bc	0.0709 (7)	0.4102 (5)	0.9208 (3)	0.043 (2)	
N11	0.33627 (4)	0.49027 (3)	0.70253 (2)	0.01528 (10)	
N12	0.48489 (4)	0.37838 (3)	0.74294 (2)	0.01563 (10)	
N13	0.30433 (4)	0.33210 (3)	0.68619 (2)	0.01395 (9)	
N14	0.32169 (4)	0.24081 (3)	0.70077 (2)	0.01745 (10)	
N15	0.23225 (4)	0.19385 (3)	0.67204 (2)	0.01868 (10)	
C11	0.38122 (4)	0.40467 (4)	0.71227 (2)	0.01382 (11)	
C12	0.40574 (5)	0.56032 (4)	0.72744 (2)	0.01632 (11)	
C13	0.51717 (5)	0.54197 (4)	0.76069 (3)	0.01833 (12)	
H13	0.5720 (6)	0.5984 (5)	0.7804 (3)	0.0363 (18)	
C14	0.55380 (5)	0.44894 (4)	0.76805 (2)	0.01681 (12)	
C15	0.35655 (6)	0.65765 (4)	0.71897 (3)	0.02196 (13)	
H15a	0.3172 (7)	0.6679 (5)	0.6726 (4)	0.050 (2)	
H15b	0.2819 (7)	0.6685 (5)	0.7483 (4)	0.047 (2)	
H15c	0.4254 (7)	0.7105 (5)	0.7310 (4)	0.052 (2)	
C16	0.67044 (6)	0.42270 (5)	0.80463 (3)	0.02361 (13)	
H16a	0.6489 (7)	0.3872 (6)	0.8456 (4)	0.057 (2)	
H16b	0.7222 (6)	0.3743 (6)	0.7798 (4)	0.052 (2)	
H16c	0.7257 (7)	0.4843 (5)	0.8169 (4)	0.051 (2)	
C17	0.20180 (5)	0.34303 (4)	0.64746 (3)	0.01577 (11)	
H17	0.1736 (6)	0.4097 (5)	0.6293 (3)	0.0366 (19)	
C18	0.15577 (4)	0.25422 (4)	0.63847 (2)	0.01561 (11)	
C19	0.04323 (5)	0.22077 (5)	0.60148 (3)	0.02058 (12)	
H19a	0.0138 (6)	0.2750 (5)	0.5675 (3)	0.0373 (19)	
H19b	0.0658 (6)	0.1571 (5)	0.5767 (3)	0.044 (2)	
C19A	−0.06241 (5)	0.20076 (5)	0.64005 (3)	0.02298 (13)	
H19c	−0.1356 (6)	0.1648 (5)	0.6122 (3)	0.0381 (19)	
H19d	−0.0301 (6)	0.1522 (5)	0.6774 (4)	0.046 (2)	
C19B	−0.11221 (7)	0.28959 (6)	0.66670 (4)	0.03246 (16)	
H19e	−0.1467 (8)	0.3395 (6)	0.6299 (4)	0.064 (3)	
H19f	−0.1855 (7)	0.2743 (6)	0.6951 (4)	0.065 (3)	
H19g	−0.0410 (7)	0.3257 (6)	0.6947 (4)	0.065 (3)	
N21	0.25418 (4)	0.15705 (3)	0.50308 (2)	0.01632 (10)	
N22	0.39878 (4)	0.26540 (3)	0.55138 (2)	0.01700 (10)	
N23	0.22387 (4)	0.31694 (3)	0.49298 (2)	0.01479 (10)	
N24	0.24824 (4)	0.40650 (3)	0.50854 (2)	0.01958 (10)	
N25	0.16744 (4)	0.45859 (3)	0.47729 (2)	0.02082 (11)	
C21	0.29765 (4)	0.24153 (4)	0.51766 (2)	0.01464 (11)	
C22	0.32269 (5)	0.08492 (4)	0.52610 (2)	0.01821 (12)	
C23	0.43087 (5)	0.10085 (4)	0.56284 (3)	0.02079 (12)	
H23	0.4850 (6)	0.0423 (5)	0.5812 (3)	0.043 (2)	
C24	0.46638 (5)	0.19313 (4)	0.57476 (3)	0.01889 (12)	
C25	0.27797 (7)	−0.01201 (5)	0.51079 (3)	0.02384 (13)	
H25a	0.1851 (7)	−0.0201 (5)	0.5205 (4)	0.060 (3)	
H25b	0.2804 (8)	−0.0262 (5)	0.4629 (4)	0.060 (3)	
H25c	0.3315 (7)	−0.0644 (5)	0.5355 (4)	0.055 (2)	
C26	0.57977 (6)	0.21713 (6)	0.61451 (3)	0.02600 (14)	
H26a	0.6343 (7)	0.2672 (6)	0.5919 (4)	0.052 (2)	
H26b	0.5564 (7)	0.2522 (6)	0.6552 (4)	0.057 (3)	
H26c	0.6347 (6)	0.1548 (6)	0.6266 (4)	0.053 (2)	
C27	0.12531 (5)	0.31185 (4)	0.45105 (3)	0.01690 (12)	
H27	0.0923 (6)	0.2461 (5)	0.4327 (3)	0.0357 (19)	
C28	0.08979 (5)	0.40339 (4)	0.44104 (3)	0.01778 (12)	
C29	−0.00879 (6)	0.44436 (5)	0.39793 (3)	0.02410 (14)	
H29a	−0.0986 (6)	0.4310 (5)	0.4141 (3)	0.047 (2)	
H29b	0.0028 (6)	0.5220 (5)	0.3986 (3)	0.041 (2)	
C29A	−0.00553 (6)	0.40846 (5)	0.33293 (3)	0.03133 (15)	
H29c	−0.0176 (7)	0.3309 (6)	0.3334 (4)	0.062 (3)	
H29d	−0.0842 (7)	0.4360 (5)	0.3056 (3)	0.054 (2)	
C29B	0.11216 (8)	0.43521 (6)	0.30568 (4)	0.03520 (17)	
H29e	0.1144 (9)	0.4047 (7)	0.2609 (4)	0.076 (3)	
H29f	0.1174 (7)	0.5125 (6)	0.3028 (4)	0.058 (2)	
H29g	0.1920 (7)	0.4100 (5)	0.3339 (3)	0.049 (2)	

### 4,6-Dimethyl-2-(4-propyl-1H-1,2,3-triazol-1-yl)pyrimidine (I) Atomic displacement parameters (Å^2^)

**Table:**

	*U*^11^	*U*^22^	*U*^33^	*U*^12^	*U*^13^	*U*^23^
N1	0.0177 (2)	0.0135 (2)	0.0184 (2)	0.00126 (18)	0.00185 (18)	0.00134 (19)
N2	0.0162 (2)	0.0165 (3)	0.0181 (2)	0.00226 (18)	−0.00106 (18)	0.00006 (19)
N3	0.0142 (2)	0.0128 (2)	0.0155 (2)	0.00086 (17)	0.00047 (17)	0.00027 (18)
N4	0.0156 (2)	0.0145 (2)	0.0194 (2)	−0.00034 (18)	−0.00083 (18)	−0.00140 (19)
N5	0.0174 (2)	0.0127 (2)	0.0209 (2)	0.00060 (18)	0.00100 (18)	−0.00058 (19)
C1	0.0153 (2)	0.0138 (3)	0.0149 (3)	0.0016 (2)	0.0009 (2)	0.0005 (2)
C2	0.0211 (3)	0.0145 (3)	0.0179 (3)	0.0031 (2)	0.0038 (2)	0.0026 (2)
C3	0.0226 (3)	0.0192 (3)	0.0201 (3)	0.0062 (3)	0.0009 (2)	0.0033 (2)
H3	0.037 (4)	0.029 (5)	0.054 (5)	0.011 (4)	−0.004 (4)	0.009 (4)
C4	0.0183 (3)	0.0205 (3)	0.0184 (3)	0.0043 (2)	−0.0006 (2)	0.0006 (2)
C5	0.0285 (3)	0.0153 (3)	0.0248 (4)	0.0019 (3)	0.0076 (3)	0.0025 (3)
H5a	0.058 (5)	0.044 (5)	0.066 (6)	0.002 (4)	0.038 (5)	0.002 (4)
H5b	0.077 (6)	0.043 (6)	0.037 (6)	−0.022 (4)	−0.002 (5)	−0.006 (4)
H5c	0.053 (5)	0.027 (5)	0.071 (6)	0.003 (4)	−0.007 (5)	0.007 (4)
C6	0.0212 (3)	0.0294 (4)	0.0310 (4)	0.0056 (3)	−0.0066 (3)	−0.0003 (3)
H6a	0.044 (5)	0.098 (8)	0.054 (6)	−0.021 (5)	−0.003 (5)	0.018 (6)
H6b	0.041 (5)	0.046 (6)	0.072 (7)	0.014 (4)	−0.015 (4)	−0.028 (5)
H6c	0.037 (5)	0.049 (6)	0.074 (7)	0.004 (4)	−0.016 (4)	0.011 (5)
C7	0.0152 (2)	0.0138 (3)	0.0170 (3)	0.0001 (2)	−0.0001 (2)	−0.0001 (2)
H7	0.030 (4)	0.022 (4)	0.042 (5)	−0.009 (3)	−0.008 (4)	−0.005 (4)
C8	0.0139 (2)	0.0146 (3)	0.0160 (3)	0.0018 (2)	0.0012 (2)	0.0011 (2)
C9	0.0165 (3)	0.0205 (4)	0.0181 (3)	0.0033 (2)	0.0014 (2)	0.0038 (3)
H9a	0.027 (4)	0.023 (5)	0.052 (5)	0.006 (3)	−0.002 (3)	0.012 (4)
H9b	0.033 (4)	0.055 (5)	0.023 (5)	0.004 (4)	0.006 (3)	−0.001 (4)
C9A	0.0154 (3)	0.0277 (4)	0.0259 (3)	0.0015 (3)	0.0013 (2)	0.0047 (3)
H9Aa	0.017 (3)	0.079 (6)	0.040 (5)	0.006 (3)	−0.005 (2)	0.023 (4)
H9Ab	0.040 (5)	0.026 (5)	0.053 (5)	−0.006 (4)	−0.001 (4)	0.000 (4)
C9B	0.0300 (4)	0.0336 (4)	0.0298 (4)	0.0094 (3)	0.0132 (3)	0.0068 (3)
H9Ba	0.107 (8)	0.046 (6)	0.063 (7)	0.032 (6)	0.033 (6)	0.006 (5)
H9Bb	0.043 (5)	0.081 (7)	0.062 (6)	0.000 (5)	0.028 (5)	0.020 (5)
H9Bc	0.044 (5)	0.055 (6)	0.029 (5)	0.000 (4)	0.004 (4)	0.000 (4)
N11	0.0159 (2)	0.0129 (2)	0.0169 (2)	0.00020 (17)	0.00094 (18)	−0.00019 (18)
N12	0.0130 (2)	0.0147 (2)	0.0189 (2)	−0.00046 (17)	−0.00039 (17)	−0.00009 (18)
N13	0.0132 (2)	0.0125 (2)	0.0159 (2)	0.00059 (17)	−0.00020 (17)	−0.00020 (18)
N14	0.0168 (2)	0.0136 (2)	0.0210 (3)	0.00023 (18)	−0.00269 (18)	0.00118 (19)
N15	0.0192 (2)	0.0133 (3)	0.0229 (3)	−0.00146 (18)	−0.00083 (19)	0.00031 (19)
C11	0.0134 (2)	0.0126 (3)	0.0154 (3)	−0.0005 (2)	0.0012 (2)	0.0002 (2)
C12	0.0197 (3)	0.0128 (3)	0.0166 (3)	−0.0014 (2)	0.0027 (2)	−0.0005 (2)
C13	0.0186 (3)	0.0163 (3)	0.0200 (3)	−0.0035 (2)	0.0011 (2)	−0.0017 (2)
H13	0.040 (4)	0.027 (4)	0.042 (5)	−0.016 (4)	0.004 (4)	−0.009 (4)
C14	0.0143 (2)	0.0168 (3)	0.0191 (3)	−0.0018 (2)	0.0006 (2)	−0.0007 (2)
C15	0.0300 (3)	0.0139 (3)	0.0218 (3)	0.0003 (3)	0.0016 (3)	−0.0007 (3)
H15a	0.079 (6)	0.029 (5)	0.040 (6)	0.019 (4)	−0.011 (5)	−0.001 (4)
H15b	0.056 (5)	0.037 (5)	0.053 (6)	0.012 (4)	0.025 (5)	0.003 (4)
H15c	0.047 (5)	0.029 (5)	0.079 (7)	−0.003 (4)	−0.007 (5)	−0.008 (4)
C16	0.0152 (3)	0.0258 (4)	0.0289 (4)	−0.0014 (3)	−0.0027 (3)	−0.0009 (3)
H16a	0.037 (5)	0.082 (7)	0.048 (6)	−0.008 (5)	−0.009 (4)	0.024 (5)
H16b	0.033 (5)	0.059 (6)	0.063 (6)	0.020 (4)	−0.008 (4)	−0.019 (5)
H16c	0.042 (5)	0.036 (5)	0.070 (6)	−0.016 (4)	−0.016 (4)	−0.007 (4)
C17	0.0147 (3)	0.0147 (3)	0.0175 (3)	0.0008 (2)	−0.0010 (2)	0.0005 (2)
H17	0.038 (4)	0.028 (5)	0.040 (5)	0.002 (4)	−0.012 (4)	0.006 (4)
C18	0.0133 (2)	0.0163 (3)	0.0171 (3)	−0.0013 (2)	0.0007 (2)	−0.0014 (2)
C19	0.0162 (3)	0.0248 (4)	0.0205 (3)	−0.0031 (2)	0.0004 (2)	−0.0048 (3)
H19a	0.032 (4)	0.047 (5)	0.031 (5)	−0.003 (4)	−0.007 (3)	0.015 (4)
H19b	0.033 (4)	0.052 (6)	0.048 (5)	−0.006 (4)	0.005 (4)	−0.030 (4)
C19A	0.0177 (3)	0.0238 (4)	0.0272 (4)	−0.0065 (3)	0.0010 (3)	−0.0005 (3)
H19c	0.025 (4)	0.035 (5)	0.055 (5)	−0.015 (3)	0.006 (4)	−0.013 (4)
H19d	0.043 (5)	0.042 (5)	0.053 (6)	−0.010 (4)	0.001 (4)	0.017 (4)
C19B	0.0206 (3)	0.0399 (4)	0.0379 (4)	−0.0057 (3)	0.0078 (3)	−0.0146 (4)
H19e	0.077 (7)	0.048 (6)	0.066 (7)	0.016 (5)	0.005 (5)	0.012 (5)
H19f	0.039 (5)	0.088 (7)	0.072 (7)	−0.020 (5)	0.029 (5)	−0.031 (5)
H19g	0.035 (5)	0.084 (7)	0.077 (7)	−0.011 (5)	0.010 (5)	−0.043 (5)
N21	0.0185 (2)	0.0140 (2)	0.0163 (2)	0.00167 (18)	0.00097 (18)	0.00152 (19)
N22	0.0151 (2)	0.0182 (3)	0.0173 (2)	0.00192 (18)	−0.00026 (18)	0.00142 (19)
N23	0.0154 (2)	0.0137 (2)	0.0150 (2)	0.00118 (17)	−0.00019 (17)	0.00144 (18)
N24	0.0214 (2)	0.0152 (3)	0.0211 (3)	−0.00049 (19)	−0.00352 (19)	0.00016 (19)
N25	0.0234 (2)	0.0134 (3)	0.0248 (3)	0.00168 (19)	−0.0021 (2)	0.0014 (2)
C21	0.0150 (2)	0.0149 (3)	0.0139 (3)	0.0015 (2)	0.0008 (2)	0.0011 (2)
C22	0.0220 (3)	0.0153 (3)	0.0176 (3)	0.0036 (2)	0.0033 (2)	0.0027 (2)
C23	0.0218 (3)	0.0198 (3)	0.0206 (3)	0.0067 (2)	0.0014 (2)	0.0041 (2)
H23	0.046 (5)	0.030 (5)	0.051 (5)	0.013 (4)	−0.001 (4)	0.017 (4)
C24	0.0156 (3)	0.0226 (3)	0.0182 (3)	0.0041 (2)	0.0002 (2)	0.0030 (2)
C25	0.0317 (3)	0.0148 (3)	0.0256 (4)	0.0020 (3)	0.0054 (3)	0.0030 (3)
H25a	0.050 (5)	0.035 (5)	0.099 (8)	−0.013 (4)	0.034 (5)	−0.017 (5)
H25b	0.114 (8)	0.042 (6)	0.027 (5)	−0.029 (5)	0.019 (5)	−0.006 (4)
H25c	0.071 (6)	0.024 (5)	0.064 (6)	0.007 (4)	−0.019 (5)	0.007 (4)
C26	0.0180 (3)	0.0332 (4)	0.0258 (4)	0.0044 (3)	−0.0031 (3)	0.0027 (3)
H26a	0.032 (4)	0.073 (7)	0.049 (6)	−0.013 (4)	−0.002 (4)	0.016 (5)
H26b	0.038 (5)	0.088 (7)	0.042 (5)	0.013 (4)	−0.010 (4)	−0.030 (5)
H26c	0.037 (5)	0.045 (5)	0.073 (6)	0.021 (4)	−0.021 (4)	−0.002 (5)
C27	0.0170 (3)	0.0146 (3)	0.0184 (3)	0.0009 (2)	−0.0018 (2)	0.0005 (2)
H27	0.030 (4)	0.026 (5)	0.050 (5)	−0.002 (3)	−0.006 (4)	−0.007 (4)
C28	0.0172 (3)	0.0158 (3)	0.0199 (3)	0.0028 (2)	−0.0002 (2)	0.0034 (2)
C29	0.0196 (3)	0.0230 (4)	0.0289 (4)	0.0047 (3)	−0.0021 (3)	0.0075 (3)
H29a	0.030 (4)	0.058 (6)	0.051 (5)	0.002 (4)	0.000 (4)	0.020 (4)
H29b	0.056 (5)	0.025 (5)	0.042 (5)	0.011 (4)	0.001 (4)	0.009 (4)
C29A	0.0377 (4)	0.0225 (4)	0.0300 (4)	0.0036 (3)	−0.0164 (3)	0.0024 (3)
H29c	0.082 (6)	0.032 (5)	0.064 (6)	−0.002 (5)	−0.032 (5)	−0.005 (4)
H29d	0.055 (4)	0.053 (5)	0.048 (5)	0.011 (3)	−0.028 (3)	0.013 (4)
C29B	0.0505 (5)	0.0330 (5)	0.0215 (4)	0.0156 (4)	0.0005 (3)	0.0024 (3)
H29e	0.113 (8)	0.090 (8)	0.026 (5)	0.031 (6)	0.008 (5)	−0.013 (5)
H29f	0.067 (6)	0.045 (6)	0.065 (7)	0.013 (5)	0.016 (5)	0.021 (5)
H29g	0.057 (5)	0.051 (6)	0.039 (5)	0.015 (4)	0.001 (4)	0.011 (4)

### 4,6-Dimethyl-2-(4-propyl-1H-1,2,3-triazol-1-yl)pyrimidine (I) Geometric parameters (Å, º)

**Table:**

N1—C1	1.3264 (7)	C15—H15c	1.083 (7)
N1—C2	1.3438 (7)	C16—H16a	1.085 (8)
N2—C1	1.3245 (6)	C16—H16b	1.079 (8)
N2—C4	1.3456 (7)	C16—H16c	1.088 (7)
N3—N4	1.3470 (6)	C17—H17	1.067 (7)
N3—C1	1.4215 (7)	C17—C18	1.3718 (8)
N3—C7	1.3610 (7)	C18—C19	1.4955 (7)
N4—N5	1.3047 (6)	C19—H19a	1.106 (7)
N5—C8	1.3683 (7)	C19—H19b	1.102 (7)
C2—C3	1.3950 (8)	C19—C19A	1.5338 (8)
C2—C5	1.4955 (8)	C19A—H19c	1.094 (6)
C3—H3	1.074 (7)	C19A—H19d	1.112 (7)
C3—C4	1.3909 (8)	C19A—C19B	1.5220 (9)
C4—C6	1.5013 (8)	C19B—H19e	1.121 (9)
C5—H5a	1.075 (7)	C19B—H19f	1.091 (8)
C5—H5b	1.090 (8)	C19B—H19g	1.081 (8)
C5—H5c	1.088 (8)	N21—C21	1.3252 (7)
C6—H6a	1.091 (9)	N21—C22	1.3464 (7)
C6—H6b	1.089 (8)	N22—C21	1.3235 (7)
C6—H6c	1.068 (8)	N22—C24	1.3455 (7)
C7—H7	1.081 (6)	N23—N24	1.3437 (6)
C7—C8	1.3740 (8)	N23—C21	1.4247 (7)
C8—C9	1.4945 (7)	N23—C27	1.3601 (7)
C9—H9a	1.112 (7)	N24—N25	1.3052 (6)
C9—H9b	1.091 (7)	N25—C28	1.3650 (7)
C9—C9A	1.5282 (8)	C22—C23	1.3943 (8)
C9A—H9Aa	1.104 (6)	C22—C25	1.4957 (8)
C9A—H9Ab	1.111 (7)	C23—H23	1.082 (7)
C9A—C9B	1.5212 (9)	C23—C24	1.3912 (8)
C9B—H9Ba	1.100 (9)	C24—C26	1.4957 (8)
C9B—H9Bb	1.097 (7)	C25—H25a	1.070 (8)
C9B—H9Bc	1.077 (8)	C25—H25b	1.083 (8)
N11—C11	1.3273 (7)	C25—H25c	1.069 (7)
N11—C12	1.3440 (7)	C26—H26a	1.086 (8)
N12—C11	1.3242 (6)	C26—H26b	1.084 (8)
N12—C14	1.3477 (7)	C26—H26c	1.094 (7)
N13—N14	1.3511 (6)	C27—H27	1.072 (7)
N13—C11	1.4236 (7)	C27—C28	1.3749 (8)
N13—C17	1.3588 (7)	C28—C29	1.4937 (8)
N14—N15	1.3044 (6)	C29—H29a	1.099 (7)
N15—C18	1.3721 (7)	C29—H29b	1.115 (7)
C12—C13	1.3927 (8)	C29—C29A	1.5327 (9)
C12—C15	1.4954 (8)	C29A—H29c	1.114 (8)
C13—H13	1.074 (6)	C29A—H29d	1.081 (7)
C13—C14	1.3920 (8)	C29A—C29B	1.5280 (11)
C14—C16	1.4976 (8)	C29B—H29e	1.087 (8)
C15—H15a	1.087 (8)	C29B—H29f	1.106 (9)
C15—H15b	1.106 (7)	C29B—H29g	1.089 (8)
			
C2—N1—C1	115.61 (5)	H16c—C16—C14	111.2 (4)
C4—N2—C1	114.89 (5)	H16c—C16—H16a	109.2 (6)
C1—N3—N4	122.04 (4)	H16c—C16—H16b	109.6 (6)
C7—N3—N4	110.84 (4)	H17—C17—N13	122.3 (4)
C7—N3—C1	127.07 (5)	C18—C17—N13	104.95 (5)
N5—N4—N3	107.11 (4)	C18—C17—H17	132.7 (4)
C8—N5—N4	109.74 (4)	C17—C18—N15	107.61 (5)
N2—C1—N1	129.31 (5)	C19—C18—N15	121.93 (5)
N3—C1—N1	114.23 (4)	C19—C18—C17	130.45 (5)
N3—C1—N2	116.46 (5)	H19a—C19—C18	108.8 (3)
C3—C2—N1	120.57 (5)	H19b—C19—C18	108.8 (3)
C5—C2—N1	117.42 (5)	H19b—C19—H19a	107.6 (6)
C5—C2—C3	122.01 (5)	C19A—C19—C18	112.63 (5)
H3—C3—C2	121.2 (4)	C19A—C19—H19a	108.8 (3)
C4—C3—C2	118.27 (5)	C19A—C19—H19b	110.1 (4)
C4—C3—H3	120.5 (4)	H19c—C19A—C19	109.2 (4)
C3—C4—N2	121.34 (5)	H19d—C19A—C19	109.0 (4)
C6—C4—N2	116.81 (5)	H19d—C19A—H19c	107.1 (5)
C6—C4—C3	121.83 (5)	C19B—C19A—C19	112.41 (6)
H5a—C5—C2	110.4 (4)	C19B—C19A—H19c	109.7 (3)
H5b—C5—C2	109.8 (4)	C19B—C19A—H19d	109.4 (4)
H5b—C5—H5a	107.5 (6)	H19e—C19B—C19A	110.8 (5)
H5c—C5—C2	111.7 (4)	H19f—C19B—C19A	111.7 (5)
H5c—C5—H5a	108.6 (6)	H19f—C19B—H19e	109.3 (6)
H5c—C5—H5b	108.8 (6)	H19g—C19B—C19A	110.6 (4)
H6a—C6—C4	110.3 (4)	H19g—C19B—H19e	106.9 (7)
H6b—C6—C4	111.5 (4)	H19g—C19B—H19f	107.4 (6)
H6b—C6—H6a	106.1 (7)	C22—N21—C21	115.29 (5)
H6c—C6—C4	111.4 (4)	C24—N22—C21	115.05 (5)
H6c—C6—H6a	106.9 (6)	C21—N23—N24	121.81 (4)
H6c—C6—H6b	110.3 (6)	C27—N23—N24	110.64 (4)
H7—C7—N3	121.4 (3)	C27—N23—C21	127.53 (5)
C8—C7—N3	104.54 (5)	N25—N24—N23	107.23 (4)
C8—C7—H7	134.0 (3)	C28—N25—N24	109.84 (4)
C7—C8—N5	107.77 (5)	N22—C21—N21	129.46 (5)
C9—C8—N5	122.21 (5)	N23—C21—N21	114.48 (4)
C9—C8—C7	130.01 (5)	N23—C21—N22	116.06 (5)
H9a—C9—C8	108.2 (3)	C23—C22—N21	120.78 (5)
H9b—C9—C8	109.3 (3)	C25—C22—N21	117.43 (5)
H9b—C9—H9a	105.7 (5)	C25—C22—C23	121.79 (5)
C9A—C9—C8	113.30 (5)	H23—C23—C22	120.1 (4)
C9A—C9—H9a	110.1 (3)	C24—C23—C22	118.25 (5)
C9A—C9—H9b	110.0 (3)	C24—C23—H23	121.6 (4)
H9Aa—C9A—C9	108.3 (3)	C23—C24—N22	121.16 (5)
H9Ab—C9A—C9	109.0 (4)	C26—C24—N22	116.74 (5)
H9Ab—C9A—H9Aa	106.8 (5)	C26—C24—C23	122.10 (5)
C9B—C9A—C9	113.28 (6)	H25a—C25—C22	110.7 (4)
C9B—C9A—H9Aa	109.9 (4)	H25b—C25—C22	110.9 (4)
C9B—C9A—H9Ab	109.3 (4)	H25b—C25—H25a	106.9 (7)
H9Ba—C9B—C9A	110.5 (4)	H25c—C25—C22	112.1 (4)
H9Bb—C9B—C9A	111.2 (5)	H25c—C25—H25a	108.1 (6)
H9Bb—C9B—H9Ba	107.9 (6)	H25c—C25—H25b	107.9 (6)
H9Bc—C9B—C9A	111.9 (4)	H26a—C26—C24	110.2 (4)
H9Bc—C9B—H9Ba	107.2 (6)	H26b—C26—C24	110.2 (4)
H9Bc—C9B—H9Bb	108.0 (6)	H26b—C26—H26a	105.4 (7)
C12—N11—C11	115.42 (4)	H26c—C26—C24	111.6 (4)
C14—N12—C11	115.01 (5)	H26c—C26—H26a	109.4 (6)
C11—N13—N14	122.62 (4)	H26c—C26—H26b	109.9 (6)
C17—N13—N14	110.57 (4)	H27—C27—N23	121.6 (4)
C17—N13—C11	126.69 (5)	C28—C27—N23	104.77 (5)
N15—N14—N13	107.19 (4)	C28—C27—H27	133.6 (4)
C18—N15—N14	109.67 (4)	C27—C28—N25	107.52 (5)
N12—C11—N11	129.26 (5)	C29—C28—N25	121.69 (5)
N13—C11—N11	113.93 (4)	C29—C28—C27	130.72 (6)
N13—C11—N12	116.81 (5)	H29a—C29—C28	110.1 (4)
C13—C12—N11	120.94 (5)	H29b—C29—C28	107.7 (4)
C15—C12—N11	117.03 (5)	H29b—C29—H29a	105.8 (5)
C15—C12—C13	122.02 (5)	C29A—C29—C28	112.93 (5)
H13—C13—C12	120.4 (4)	C29A—C29—H29a	110.5 (4)
C14—C13—C12	118.12 (5)	C29A—C29—H29b	109.4 (4)
C14—C13—H13	121.5 (4)	H29c—C29A—C29	108.0 (4)
C13—C14—N12	121.25 (5)	H29d—C29A—C29	108.3 (4)
C16—C14—N12	117.08 (5)	H29d—C29A—H29c	106.1 (6)
C16—C14—C13	121.66 (5)	C29B—C29A—C29	112.65 (6)
H15a—C15—C12	110.5 (4)	C29B—C29A—H29c	111.0 (5)
H15b—C15—C12	109.7 (4)	C29B—C29A—H29d	110.5 (4)
H15b—C15—H15a	106.7 (6)	H29e—C29B—C29A	110.8 (5)
H15c—C15—C12	112.3 (4)	H29f—C29B—C29A	108.8 (4)
H15c—C15—H15a	110.1 (6)	H29f—C29B—H29e	109.9 (7)
H15c—C15—H15b	107.4 (6)	H29g—C29B—C29A	110.9 (4)
H16a—C16—C14	109.0 (4)	H29g—C29B—H29e	107.7 (6)
H16b—C16—C14	110.5 (4)	H29g—C29B—H29f	108.6 (6)
H16b—C16—H16a	107.2 (7)		
			
N1—C1—N2—C4	−0.78 (7)	N13—C17—C18—N15	0.00 (5)
N1—C1—N3—N4	170.06 (4)	N13—C17—C18—C19	178.66 (4)
N1—C1—N3—C7	−7.24 (6)	N14—N15—C18—C17	0.01 (5)
N1—C2—C3—C4	−0.44 (6)	N14—N15—C18—C19	−178.78 (4)
N2—C1—N3—N4	−9.83 (6)	N15—C18—C19—C19A	77.84 (6)
N2—C1—N3—C7	172.87 (4)	C12—C13—C14—C16	−178.18 (5)
N2—C4—C3—C2	−0.02 (6)	C17—C18—C19—C19A	−100.65 (7)
N3—N4—N5—C8	−0.08 (4)	C18—C19—C19A—C19B	68.29 (6)
N3—C7—C8—N5	0.01 (5)	N21—C21—N22—C24	−0.85 (7)
N3—C7—C8—C9	−179.65 (4)	N21—C21—N23—N24	172.88 (4)
N4—N5—C8—C7	0.05 (5)	N21—C21—N23—C27	−9.13 (6)
N4—N5—C8—C9	179.73 (4)	N21—C22—C23—C24	−0.36 (6)
N5—C8—C9—C9A	−120.86 (6)	N22—C21—N23—N24	−7.25 (6)
C2—C3—C4—C6	−178.52 (6)	N22—C21—N23—C27	170.74 (4)
C7—C8—C9—C9A	58.75 (7)	N22—C24—C23—C22	−0.27 (6)
C8—C9—C9A—C9B	66.23 (6)	N23—N24—N25—C28	−0.06 (5)
N11—C11—N12—C14	0.45 (7)	N23—C27—C28—N25	−0.32 (5)
N11—C11—N13—N14	−166.64 (4)	N23—C27—C28—C29	176.70 (4)
N11—C11—N13—C17	8.93 (6)	N24—N25—C28—C27	0.24 (5)
N11—C12—C13—C14	−0.82 (6)	N24—N25—C28—C29	−177.10 (5)
N12—C11—N13—N14	12.55 (6)	N25—C28—C29—C29A	127.01 (6)
N12—C11—N13—C17	−171.88 (4)	C22—C23—C24—C26	178.92 (5)
N12—C14—C13—C12	1.15 (6)	C27—C28—C29—C29A	−49.65 (7)
N13—N14—N15—C18	−0.02 (5)	C28—C29—C29A—C29B	−62.73 (6)

### 4,6-Dimethyl-2-(4-propyl-1H-1,2,3-triazol-1-yl)pyrimidine (I) Hydrogen-bond geometry (Å, º)

**Table:**

*D*—H···*A*	*D*—H	H···*A*	*D*···*A*	*D*—H···*A*
C3—H3···N24^i^	1.074 (7)	2.279 (7)	3.3359 (8)	167.3 (5)
C3—H3···N25^i^	1.074 (7)	2.519 (7)	3.5487 (8)	160.2 (5)
C5—H5*a*···N25^ii^	1.075 (7)	2.641 (7)	3.6399 (8)	154.2 (6)
C13—H13···N14^iii^	1.074 (6)	2.361 (7)	3.4097 (8)	165.1 (5)
C15—H15*c*···N12^iii^	1.083 (7)	2.634 (8)	3.6588 (8)	157.7 (5)
C23—H23···N4^i^	1.082 (7)	2.300 (7)	3.3753 (8)	172.2 (6)
C23—H23···N5^i^	1.082 (7)	2.629 (7)	3.6525 (7)	157.6 (5)
C25—H25*b*···N5^iv^	1.083 (8)	2.520 (8)	3.4538 (9)	143.8 (5)

Symmetry codes: (i) −*x*+1, *y*−1/2, −*z*+3/2; (ii) *x*, −*y*+1/2, *z*+1/2; (iii) −*x*+1, *y*+1/2, −*z*+3/2; (iv) *x*, −*y*+1/2, *z*−1/2.

### Di-µ-iodido-bis{[4,6-dimethyl-2-(4-propyl-1H-1,2,3-triazol-1-yl-κN2)pyrimidine-κN1]copper(I)} (II) Crystal data

**Table:**

[Cu_2_I_2_(C_11_H_15_N_5_)_2_]	*F*(000) = 1584.480
*M**_r_* = 815.45	*D*_x_ = 1.893 Mg m^−^^3^
Monoclinic, *P*2_1_/*n*	Mo *K*α radiation, λ = 0.71073 Å
*a* = 11.5497 (3) Å	Cell parameters from 54960 reflections
*b* = 18.2906 (3) Å	θ = 2.5–38.0°
*c* = 13.7287 (3) Å	µ = 3.68 mm^−^^1^
β = 99.489 (2)°	*T* = 100 K
*V* = 2860.54 (10) Å^3^	Prism, clear dark yellow
*Z* = 4	0.23 × 0.11 × 0.09 mm

### Di-µ-iodido-bis{[4,6-dimethyl-2-(4-propyl-1H-1,2,3-triazol-1-yl-κN2)pyrimidine-κN1]copper(I)} (II) Data collection

**Table:**

SuperNova, Dual, Cu at home/near, Pilatus 200K diffractometer	8347 independent reflections
Radiation source: micro-focus sealed X-ray tube, SuperNova (Mo) X-ray Source	7323 reflections with *I*≥ 2u(*I*)
Mirror monochromator	*R*_int_ = 0.060
Detector resolution: 5.8140 pixels mm^-1^	θ_max_ = 30.0°, θ_min_ = 2.2°
ω scans	*h* = −19→19
Absorption correction: multi-scan (CrysAlisPro; Rigaku OD, 2024	*k* = −30→31
*T*_min_ = 0.745, *T*_max_ = 1.000	*l* = −23→23
142732 measured reflections	

### Di-µ-iodido-bis{[4,6-dimethyl-2-(4-propyl-1H-1,2,3-triazol-1-yl-κN2)pyrimidine-κN1]copper(I)} (II) Refinement

**Table:**

Refinement on *F*^2^	0 constraints
Least-squares matrix: full	Primary atom site location: dual
*R*[*F*^2^ > 2σ(*F*^2^)] = 0.019	All H-atom parameters refined
*wR*(*F*^2^) = 0.037	*w* = 1/[σ^2^(*F*_o_^2^) + (0.0047*P*)^2^ + 2.3241*P*] where *P* = (*F*_o_^2^ + 2*F*_c_^2^)/3
*S* = 1.02	(Δ/σ)_max_ = 0.0004
8347 reflections	Δρ_max_ = 0.84 e Å^−^^3^
595 parameters	Δρ_min_ = −0.64 e Å^−^^3^
189 restraints	

### Di-µ-iodido-bis{[4,6-dimethyl-2-(4-propyl-1H-1,2,3-triazol-1-yl-κN2)pyrimidine-κN1]copper(I)} (II) Fractional atomic coordinates and isotropic or equivalent isotropic displacement parameters (Å^2^)

**Table:**

	*x*	*y*	*z*	*U*_iso_*/*U*_eq_	
I1	0.171709 (10)	0.590489 (6)	0.218744 (8)	0.01963 (3)	
I2	0.556934 (10)	0.562985 (6)	0.257338 (7)	0.01722 (3)	
Cu1	0.380823 (19)	0.641588 (11)	0.287628 (15)	0.01717 (4)	
Cu2	0.348437 (19)	0.504984 (11)	0.251061 (15)	0.01706 (4)	
N1	0.37958 (12)	0.68975 (7)	0.42942 (9)	0.0153 (3)	
N2	0.38237 (12)	0.80767 (7)	0.50545 (10)	0.0159 (3)	
N3	0.40934 (12)	0.79459 (7)	0.34228 (9)	0.0142 (3)	
N4	0.41459 (12)	0.75164 (7)	0.26307 (9)	0.0147 (3)	
N5	0.43706 (12)	0.79350 (7)	0.19177 (10)	0.0157 (3)	
N11	0.35681 (12)	0.41514 (7)	0.15537 (9)	0.0148 (3)	
N12	0.40624 (13)	0.28818 (7)	0.16746 (10)	0.0163 (3)	
N13	0.35913 (12)	0.35205 (7)	0.30173 (9)	0.0137 (3)	
N14	0.34501 (13)	0.41641 (7)	0.34709 (10)	0.0155 (3)	
N15	0.32523 (13)	0.40190 (7)	0.43560 (10)	0.0160 (3)	
C1	0.38900 (14)	0.76219 (8)	0.43197 (11)	0.0139 (3)	
C2	0.36126 (15)	0.65751 (9)	0.51410 (11)	0.0168 (3)	
C3	0.35182 (15)	0.70027 (9)	0.59639 (12)	0.0185 (3)	
H3	0.336 (2)	0.6748 (12)	0.6632 (14)	0.039 (6)	
C4	0.36262 (15)	0.77626 (9)	0.58987 (11)	0.0175 (3)	
C5	0.3507 (2)	0.57632 (10)	0.51299 (14)	0.0240 (4)	
H5a	0.275 (2)	0.5610 (13)	0.461 (2)	0.056 (8)	
H5b	0.424 (2)	0.5504 (13)	0.4896 (19)	0.055 (7)	
H5c	0.345 (2)	0.5572 (13)	0.5840 (18)	0.056 (7)	
C6	0.35362 (19)	0.82511 (11)	0.67540 (14)	0.0239 (4)	
H6a	0.426 (3)	0.8158 (17)	0.733 (2)	0.080 (9)	
H6b	0.347 (3)	0.8789 (14)	0.6526 (18)	0.063 (8)	
H6c	0.273 (2)	0.8147 (15)	0.7058 (19)	0.060 (8)	
C7	0.42815 (15)	0.86591 (9)	0.32041 (12)	0.0157 (3)	
H7	0.427 (2)	0.9083 (11)	0.3737 (14)	0.031 (6)	
C8	0.44547 (14)	0.86444 (8)	0.22353 (11)	0.0149 (3)	
C9	0.47053 (16)	0.92537 (9)	0.15763 (12)	0.0162 (3)	
H9a	0.470 (2)	0.9043 (12)	0.0849 (17)	0.048 (7)	
H9b	0.4004 (18)	0.9648 (11)	0.1508 (15)	0.029 (5)	
C9A	0.58566 (18)	0.96549 (11)	0.19265 (15)	0.0258 (4)	
H9Aa	0.589 (2)	0.9851 (15)	0.2655 (19)	0.063 (8)	
H9Ab	0.657 (2)	0.9280 (13)	0.1987 (19)	0.052 (7)	
C9B	0.60358 (19)	1.02840 (12)	0.12462 (16)	0.0279 (4)	
H9Ba	0.606 (2)	1.0080 (13)	0.0533 (18)	0.049 (7)	
H9Bb	0.530 (2)	1.0652 (13)	0.1184 (17)	0.047 (7)	
H9Bc	0.682 (2)	1.0570 (14)	0.1489 (18)	0.060 (7)	
C11	0.37574 (14)	0.35126 (8)	0.20203 (11)	0.0135 (3)	
C12	0.37085 (15)	0.41590 (9)	0.05974 (11)	0.0166 (3)	
C13	0.40504 (15)	0.35250 (9)	0.01560 (12)	0.0183 (3)	
H13	0.416 (2)	0.3524 (11)	−0.0606 (14)	0.032 (5)	
C14	0.42089 (15)	0.28856 (9)	0.07192 (12)	0.0168 (3)	
C15	0.3447 (2)	0.48637 (10)	0.00552 (13)	0.0235 (4)	
H15a	0.252 (2)	0.5008 (14)	0.0032 (19)	0.055 (8)	
H15b	0.399 (2)	0.5306 (14)	0.0437 (19)	0.056 (8)	
H15c	0.363 (2)	0.4833 (13)	−0.0677 (18)	0.057 (7)	
C16	0.45449 (19)	0.21749 (10)	0.03065 (14)	0.0227 (4)	
H16a	0.535 (3)	0.1954 (16)	0.076 (2)	0.078 (10)	
H16b	0.393 (2)	0.1748 (15)	0.041 (2)	0.061 (8)	
H16c	0.460 (3)	0.2200 (15)	−0.043 (2)	0.079 (9)	
C17	0.34799 (14)	0.29500 (9)	0.36310 (11)	0.0146 (3)	
H17	0.353 (2)	0.2381 (11)	0.3401 (15)	0.029 (5)	
C18	0.32579 (14)	0.32746 (8)	0.44871 (11)	0.0141 (3)	
C19	0.30075 (16)	0.29312 (9)	0.54173 (12)	0.0161 (3)	
H19a	0.376 (2)	0.3022 (11)	0.5999 (14)	0.033 (6)	
H19b	0.228 (2)	0.3230 (12)	0.5632 (15)	0.036 (6)	
C19A	0.27294 (18)	0.21167 (9)	0.53175 (13)	0.0202 (3)	
H19c	0.197 (2)	0.2044 (11)	0.4717 (16)	0.036 (6)	
H19d	0.3472 (19)	0.1811 (11)	0.5102 (15)	0.031 (5)	
C19B	0.24583 (19)	0.17938 (11)	0.62792 (13)	0.0236 (4)	
H19e	0.231 (2)	0.1214 (13)	0.6230 (16)	0.048 (7)	
H19f	0.320 (2)	0.1875 (13)	0.6886 (16)	0.047 (7)	
H19g	0.171 (2)	0.2040 (13)	0.6494 (18)	0.051 (7)	

### Di-µ-iodido-bis{[4,6-dimethyl-2-(4-propyl-1H-1,2,3-triazol-1-yl-κN2)pyrimidine-κN1]copper(I)} (II) Atomic displacement parameters (Å^2^)

**Table:**

	*U*^11^	*U*^22^	*U*^33^	*U*^12^	*U*^13^	*U*^23^
I1	0.01699 (6)	0.01271 (5)	0.02915 (6)	0.00027 (4)	0.00363 (4)	0.00414 (4)
I2	0.01696 (5)	0.01709 (5)	0.01794 (5)	−0.00039 (4)	0.00388 (4)	−0.00046 (4)
Cu1	0.02222 (11)	0.01073 (9)	0.01926 (9)	−0.00160 (8)	0.00550 (8)	−0.00065 (7)
Cu2	0.02156 (11)	0.01016 (9)	0.01982 (9)	0.00018 (8)	0.00452 (8)	−0.00016 (7)
N1	0.0188 (7)	0.0128 (6)	0.0147 (6)	−0.0017 (5)	0.0043 (5)	0.0021 (5)
N2	0.0182 (7)	0.0150 (7)	0.0152 (6)	−0.0008 (5)	0.0044 (5)	0.0000 (5)
N3	0.0190 (7)	0.0104 (6)	0.0138 (6)	−0.0001 (5)	0.0042 (5)	0.0008 (5)
N4	0.0209 (7)	0.0096 (6)	0.0143 (6)	−0.0013 (5)	0.0051 (5)	0.0009 (5)
N5	0.0209 (7)	0.0112 (6)	0.0160 (6)	−0.0001 (5)	0.0061 (5)	0.0010 (5)
N11	0.0193 (7)	0.0112 (6)	0.0143 (6)	0.0007 (5)	0.0039 (5)	0.0004 (5)
N12	0.0204 (7)	0.0117 (6)	0.0176 (6)	0.0015 (5)	0.0059 (5)	−0.0002 (5)
N13	0.0178 (7)	0.0098 (6)	0.0140 (6)	0.0002 (5)	0.0044 (5)	−0.0012 (5)
N14	0.0212 (7)	0.0099 (6)	0.0157 (6)	−0.0009 (5)	0.0040 (5)	−0.0001 (5)
N15	0.0223 (7)	0.0108 (6)	0.0154 (6)	−0.0007 (5)	0.0041 (5)	−0.0014 (5)
C1	0.0154 (8)	0.0128 (7)	0.0142 (7)	−0.0001 (6)	0.0043 (6)	0.0010 (5)
C2	0.0194 (8)	0.0158 (8)	0.0152 (7)	−0.0017 (6)	0.0034 (6)	0.0032 (6)
C3	0.0220 (9)	0.0196 (8)	0.0151 (7)	−0.0014 (7)	0.0066 (6)	0.0031 (6)
H3	0.061 (14)	0.036 (11)	0.022 (6)	−0.005 (7)	0.017 (4)	0.008 (4)
C4	0.0191 (8)	0.0203 (8)	0.0140 (7)	−0.0013 (6)	0.0054 (6)	−0.0013 (6)
C5	0.0353 (12)	0.0156 (8)	0.0218 (8)	−0.0021 (8)	0.0069 (8)	0.0052 (7)
H5a	0.061 (15)	0.036 (13)	0.068 (14)	−0.014 (8)	0.001 (8)	0.002 (8)
H5b	0.060 (15)	0.035 (13)	0.072 (14)	0.005 (8)	0.020 (8)	0.005 (8)
H5c	0.073 (15)	0.057 (14)	0.045 (12)	−0.003 (8)	0.026 (8)	0.013 (8)
C6	0.0300 (11)	0.0245 (9)	0.0186 (8)	−0.0007 (8)	0.0081 (8)	−0.0035 (7)
H6a	0.085 (17)	0.097 (17)	0.053 (14)	0.016 (9)	0.000 (8)	−0.012 (9)
H6b	0.099 (17)	0.048 (13)	0.046 (13)	0.000 (9)	0.018 (8)	−0.004 (8)
H6c	0.055 (14)	0.070 (15)	0.061 (14)	−0.016 (8)	0.030 (8)	−0.015 (8)
C7	0.0207 (8)	0.0109 (7)	0.0167 (7)	−0.0003 (6)	0.0064 (6)	−0.0003 (6)
H7	0.045 (13)	0.028 (11)	0.022 (10)	0.001 (7)	0.006 (7)	−0.005 (7)
C8	0.0193 (8)	0.0105 (7)	0.0153 (7)	0.0008 (6)	0.0044 (6)	0.0014 (5)
C9	0.0195 (9)	0.0117 (7)	0.0179 (7)	−0.0005 (6)	0.0042 (6)	0.0020 (6)
H9a	0.061 (14)	0.041 (12)	0.042 (12)	−0.014 (8)	0.002 (8)	−0.001 (8)
H9b	0.032 (12)	0.023 (11)	0.035 (11)	0.006 (7)	0.012 (7)	0.010 (7)
C9A	0.0211 (10)	0.0240 (10)	0.0313 (10)	−0.0050 (8)	0.0013 (8)	0.0154 (8)
H9Aa	0.068 (15)	0.070 (15)	0.050 (13)	−0.004 (8)	0.004 (8)	0.005 (8)
H9Ab	0.045 (13)	0.042 (13)	0.071 (14)	0.006 (8)	0.017 (8)	0.022 (8)
C9B	0.0236 (11)	0.0259 (10)	0.0338 (10)	−0.0058 (8)	0.0033 (8)	0.0138 (8)
H9Ba	0.062 (14)	0.046 (13)	0.045 (12)	0.001 (8)	0.030 (8)	0.003 (8)
H9Bb	0.042 (13)	0.048 (13)	0.048 (13)	0.006 (8)	0.002 (8)	0.004 (8)
H9Bc	0.058 (14)	0.058 (14)	0.059 (14)	−0.021 (8)	0.001 (8)	0.013 (8)
C11	0.0150 (7)	0.0109 (7)	0.0152 (7)	−0.0007 (6)	0.0040 (6)	−0.0010 (5)
C12	0.0226 (9)	0.0137 (7)	0.0139 (7)	0.0000 (6)	0.0040 (6)	0.0008 (6)
C13	0.0235 (9)	0.0170 (8)	0.0153 (7)	0.0008 (7)	0.0056 (6)	−0.0015 (6)
H13	0.052 (13)	0.029 (11)	0.018 (4)	0.009 (7)	0.013 (3)	0.000 (3)
C14	0.0197 (8)	0.0141 (7)	0.0174 (7)	0.0008 (6)	0.0059 (6)	−0.0029 (6)
C15	0.0350 (11)	0.0188 (9)	0.0174 (8)	0.0034 (8)	0.0065 (8)	0.0046 (7)
H15a	0.054 (14)	0.054 (14)	0.063 (14)	0.013 (8)	0.026 (8)	0.010 (8)
H15b	0.067 (15)	0.033 (13)	0.067 (14)	−0.008 (8)	0.010 (8)	0.007 (8)
H15c	0.086 (16)	0.049 (13)	0.041 (12)	0.010 (8)	0.022 (8)	0.003 (8)
C16	0.0291 (11)	0.0189 (9)	0.0216 (8)	0.0026 (8)	0.0088 (8)	−0.0045 (7)
H16a	0.072 (16)	0.077 (16)	0.079 (16)	0.010 (9)	−0.003 (9)	−0.013 (9)
H16b	0.047 (14)	0.049 (14)	0.088 (15)	−0.001 (8)	0.019 (8)	−0.017 (8)
H16c	0.121 (18)	0.063 (15)	0.057 (13)	0.022 (9)	0.024 (9)	−0.001 (8)
C17	0.0176 (8)	0.0120 (7)	0.0145 (7)	−0.0001 (6)	0.0038 (6)	0.0002 (6)
H17	0.047 (13)	0.012 (5)	0.027 (10)	0.004 (3)	0.007 (7)	−0.002 (3)
C18	0.0167 (8)	0.0107 (7)	0.0152 (7)	−0.0007 (6)	0.0037 (6)	−0.0003 (5)
C19	0.0193 (9)	0.0145 (8)	0.0152 (7)	−0.0010 (6)	0.0048 (6)	−0.0001 (6)
H19a	0.043 (13)	0.035 (12)	0.018 (10)	−0.011 (8)	0.003 (7)	0.003 (7)
H19b	0.042 (13)	0.041 (12)	0.028 (11)	0.003 (8)	0.017 (7)	0.001 (7)
C19A	0.0289 (10)	0.0141 (8)	0.0183 (8)	−0.0006 (7)	0.0058 (7)	0.0018 (6)
H19c	0.043 (13)	0.027 (11)	0.036 (11)	−0.012 (7)	0.003 (8)	0.009 (7)
H19d	0.037 (12)	0.026 (11)	0.031 (11)	0.007 (7)	0.008 (7)	0.002 (7)
C19B	0.0320 (11)	0.0196 (9)	0.0201 (8)	−0.0003 (8)	0.0065 (8)	0.0059 (7)
H19e	0.062 (14)	0.044 (13)	0.038 (12)	−0.010 (8)	0.013 (8)	0.005 (8)
H19f	0.041 (13)	0.065 (14)	0.033 (11)	0.001 (8)	−0.001 (8)	−0.004 (8)
H19g	0.044 (13)	0.053 (13)	0.060 (13)	0.007 (8)	0.022 (8)	−0.001 (8)

### Di-µ-iodido-bis{[4,6-dimethyl-2-(4-propyl-1H-1,2,3-triazol-1-yl-κN2)pyrimidine-κN1]copper(I)} (II) Geometric parameters (Å, º)

**Table:**

Cu1—I1	2.6169 (2)	C6—H6c	1.10 (3)
Cu2—I1	2.5516 (2)	C7—H7	1.07 (2)
Cu1—I2	2.5800 (2)	C7—C8	1.377 (2)
Cu2—I2	2.6198 (2)	C8—C9	1.494 (2)
Cu1—Cu2	2.5638 (3)	C9—H9a	1.07 (2)
Cu1—N1	2.1388 (13)	C9—H9b	1.08 (2)
Cu1—N4	2.0883 (13)	C9—C9A	1.525 (3)
Cu2—N11	2.1160 (13)	C9A—H9Aa	1.06 (3)
Cu2—N14	2.0932 (13)	C9A—H9Ab	1.06 (2)
N1—C1	1.3294 (19)	C9A—C9B	1.518 (2)
N1—C2	1.3511 (19)	C9B—H9Ba	1.05 (2)
N2—C1	1.3194 (19)	C9B—H9Bb	1.07 (2)
N2—C4	1.346 (2)	C9B—H9Bc	1.05 (3)
N3—N4	1.3509 (17)	C12—C13	1.395 (2)
N3—C1	1.4205 (19)	C12—C15	1.494 (2)
N3—C7	1.3640 (19)	C13—H13	1.075 (19)
N4—N5	1.3021 (17)	C13—C14	1.397 (2)
N5—C8	1.3672 (19)	C14—C16	1.495 (2)
N11—C11	1.3331 (19)	C15—H15a	1.10 (3)
N11—C12	1.3491 (19)	C15—H15b	1.10 (3)
N12—C11	1.3174 (19)	C15—H15c	1.06 (2)
N12—C14	1.350 (2)	C16—H16a	1.11 (3)
N13—N14	1.3540 (17)	C16—H16b	1.08 (3)
N13—C11	1.4132 (19)	C16—H16c	1.03 (3)
N13—C17	1.3605 (19)	C17—H17	1.092 (19)
N14—N15	1.3003 (18)	C17—C18	1.378 (2)
N15—C18	1.3733 (19)	C18—C19	1.493 (2)
C2—C3	1.393 (2)	C19—H19a	1.09 (2)
C2—C5	1.490 (2)	C19—H19b	1.08 (2)
C3—H3	1.070 (19)	C19—C19A	1.525 (2)
C3—C4	1.400 (2)	C19A—H19c	1.11 (2)
C4—C6	1.493 (2)	C19A—H19d	1.10 (2)
C5—H5a	1.07 (3)	C19A—C19B	1.525 (2)
C5—H5b	1.06 (3)	C19B—H19e	1.07 (2)
C5—H5c	1.05 (2)	C19B—H19f	1.10 (2)
C6—H6a	1.07 (3)	C19B—H19g	1.05 (2)
C6—H6b	1.03 (3)		
			
Cu2—I1—Cu1	59.461 (7)	C8—C7—H7	134.1 (11)
Cu2—I2—Cu1	59.077 (7)	C7—C8—N5	108.21 (14)
I2—Cu1—I1	116.610 (8)	C9—C8—N5	121.71 (14)
Cu2—Cu1—I1	59.002 (7)	C9—C8—C7	130.08 (14)
Cu2—Cu1—I2	61.236 (7)	H9a—C9—C8	109.0 (12)
N1—Cu1—I1	109.37 (4)	H9b—C9—C8	109.5 (11)
N1—Cu1—I2	120.00 (4)	H9b—C9—H9a	105.8 (17)
N1—Cu1—Cu2	123.85 (4)	C9A—C9—C8	114.45 (14)
N4—Cu1—I1	118.15 (4)	C9A—C9—H9a	109.7 (13)
N4—Cu1—I2	109.75 (4)	C9A—C9—H9b	108.0 (11)
N4—Cu1—Cu2	158.89 (4)	H9Aa—C9A—C9	110.8 (15)
N4—Cu1—N1	77.25 (5)	H9Ab—C9A—C9	109.6 (13)
I2—Cu2—I1	117.526 (8)	H9Ab—C9A—H9Aa	103.8 (19)
Cu1—Cu2—I1	61.537 (7)	C9B—C9A—C9	111.78 (15)
Cu1—Cu2—I2	59.686 (7)	C9B—C9A—H9Aa	109.7 (15)
N11—Cu2—I1	119.02 (4)	C9B—C9A—H9Ab	110.9 (13)
N11—Cu2—I2	101.48 (4)	H9Ba—C9B—C9A	109.3 (13)
N11—Cu2—Cu1	149.13 (4)	H9Bb—C9B—C9A	109.8 (13)
N14—Cu2—I1	119.10 (4)	H9Bb—C9B—H9Ba	106.7 (18)
N14—Cu2—I2	113.92 (4)	H9Bc—C9B—C9A	112.3 (13)
N14—Cu2—Cu1	130.41 (4)	H9Bc—C9B—H9Ba	108.7 (19)
N14—Cu2—N11	78.32 (5)	H9Bc—C9B—H9Bb	109.9 (19)
C1—N1—Cu1	114.94 (10)	N12—C11—N11	128.82 (14)
C2—N1—Cu1	128.97 (11)	N13—C11—N11	114.75 (13)
C2—N1—C1	115.86 (14)	N13—C11—N12	116.42 (13)
C4—N2—C1	115.38 (14)	C13—C12—N11	120.14 (14)
C1—N3—N4	119.40 (12)	C15—C12—N11	116.54 (15)
C7—N3—N4	110.42 (12)	C15—C12—C13	123.29 (15)
C7—N3—C1	130.16 (13)	H13—C13—C12	120.8 (11)
N3—N4—Cu1	113.34 (9)	C14—C13—C12	118.41 (15)
N5—N4—Cu1	138.92 (10)	C14—C13—H13	120.7 (11)
N5—N4—N3	107.71 (12)	C13—C14—N12	121.06 (15)
C8—N5—N4	109.31 (13)	C16—C14—N12	116.48 (15)
C11—N11—Cu2	113.76 (10)	C16—C14—C13	122.46 (15)
C12—N11—Cu2	128.41 (11)	H15a—C15—C12	109.8 (13)
C12—N11—C11	116.11 (14)	H15b—C15—C12	110.1 (13)
C14—N12—C11	115.43 (14)	H15b—C15—H15a	108.1 (19)
C11—N13—N14	120.01 (12)	H15c—C15—C12	111.7 (13)
C17—N13—N14	110.56 (12)	H15c—C15—H15a	109.3 (19)
C17—N13—C11	129.33 (13)	H15c—C15—H15b	108 (2)
N13—N14—Cu2	111.53 (9)	H16a—C16—C14	110.4 (15)
N15—N14—Cu2	140.45 (11)	H16b—C16—C14	110.9 (14)
N15—N14—N13	107.78 (12)	H16b—C16—H16a	100 (2)
C18—N15—N14	109.10 (13)	H16c—C16—C14	113.4 (15)
N2—C1—N1	129.32 (14)	H16c—C16—H16a	113 (2)
N3—C1—N1	114.68 (13)	H16c—C16—H16b	108 (2)
N3—C1—N2	116.01 (13)	H17—C17—N13	122.4 (11)
C3—C2—N1	119.81 (15)	C18—C17—N13	104.34 (13)
C5—C2—N1	116.76 (15)	C18—C17—H17	133.2 (11)
C5—C2—C3	123.42 (15)	C17—C18—N15	108.22 (13)
H3—C3—C2	119.8 (12)	C19—C18—N15	122.12 (14)
C4—C3—C2	119.08 (15)	C19—C18—C17	129.62 (14)
C4—C3—H3	121.1 (12)	H19a—C19—C18	108.7 (11)
C3—C4—N2	120.54 (15)	H19b—C19—C18	106.8 (11)
C6—C4—N2	117.74 (15)	H19b—C19—H19a	106.3 (16)
C6—C4—C3	121.72 (15)	C19A—C19—C18	113.67 (13)
H5a—C5—C2	108.9 (13)	C19A—C19—H19a	110.2 (11)
H5b—C5—C2	112.4 (13)	C19A—C19—H19b	110.9 (12)
H5b—C5—H5a	107 (2)	H19c—C19A—C19	108.3 (11)
H5c—C5—C2	109.9 (14)	H19d—C19A—C19	110.9 (11)
H5c—C5—H5a	111 (2)	H19d—C19A—H19c	107.0 (16)
H5c—C5—H5b	108.1 (19)	C19B—C19A—C19	111.76 (15)
H6a—C6—C4	110.1 (16)	C19B—C19A—H19c	110.4 (11)
H6b—C6—C4	110.1 (14)	C19B—C19A—H19d	108.4 (10)
H6b—C6—H6a	113 (2)	H19e—C19B—C19A	112.3 (12)
H6c—C6—C4	111.6 (13)	H19f—C19B—C19A	110.8 (13)
H6c—C6—H6a	107 (2)	H19f—C19B—H19e	106.0 (18)
H6c—C6—H6b	105 (2)	H19g—C19B—C19A	111.7 (13)
H7—C7—N3	121.6 (11)	H19g—C19B—H19e	108.2 (18)
C8—C7—N3	104.34 (14)	H19g—C19B—H19f	107.5 (18)
			
Cu1—N1—C1—N2	174.49 (11)	N11—C11—N13—C17	165.35 (14)
Cu1—N1—C1—N3	−5.63 (11)	N11—C12—C13—C14	1.74 (19)
Cu1—N1—C2—C3	−173.21 (14)	N12—C11—N11—C12	−0.2 (2)
Cu1—N1—C2—C5	6.07 (16)	N12—C11—N13—N14	170.16 (14)
Cu1—N4—N3—C1	3.36 (13)	N12—C11—N13—C17	−13.9 (2)
Cu1—N4—N3—C7	−177.92 (11)	N12—C14—C13—C12	−1.51 (19)
Cu1—N4—N5—C8	177.11 (17)	N13—N14—N15—C18	−0.26 (14)
Cu2—N11—C11—N12	−166.53 (11)	N13—C11—N11—C12	−179.37 (14)
Cu2—N11—C11—N13	14.28 (11)	N13—C11—N12—C14	179.59 (14)
Cu2—N11—C12—C13	163.07 (14)	N13—C17—C18—N15	−0.36 (14)
Cu2—N11—C12—C15	−18.62 (16)	N13—C17—C18—C19	177.28 (12)
Cu2—N14—N13—C11	1.10 (13)	N14—N13—C17—C18	0.21 (14)
Cu2—N14—N13—C17	−175.51 (11)	N14—N15—C18—C17	0.40 (15)
Cu2—N14—N15—C18	173.21 (18)	N14—N15—C18—C19	−177.46 (13)
N1—C1—N2—C4	−0.4 (2)	N15—N14—N13—C11	176.64 (12)
N1—C1—N3—N4	1.58 (17)	N15—N14—N13—C17	0.02 (15)
N1—C1—N3—C7	−176.86 (14)	N15—C18—C19—C19A	164.29 (17)
N1—C2—C3—C4	−0.56 (19)	C1—N1—C2—C3	0.89 (17)
N2—C1—N1—C2	−0.4 (2)	C1—N1—C2—C5	−179.84 (16)
N2—C1—N3—N4	−178.53 (14)	C1—N2—C4—C3	0.72 (17)
N2—C1—N3—C7	3.0 (2)	C1—N2—C4—C6	−179.68 (16)
N2—C4—C3—C2	−0.29 (19)	C1—N3—C7—C8	178.43 (19)
N3—N4—N5—C8	−0.64 (14)	C2—C3—C4—C6	−179.87 (17)
N3—C1—N1—C2	179.43 (14)	C4—C3—C2—C5	−179.78 (17)
N3—C1—N2—C4	179.75 (14)	C7—C8—C9—C9A	62.4 (2)
N3—C7—C8—N5	−0.27 (15)	C8—C9—C9A—C9B	−177.87 (18)
N3—C7—C8—C9	−179.79 (12)	C11—N11—C12—C13	−0.93 (17)
N4—N3—C7—C8	−0.11 (14)	C11—N11—C12—C15	177.37 (16)
N4—N5—C8—C7	0.58 (15)	C11—N12—C14—C13	0.47 (18)
N4—N5—C8—C9	−179.86 (13)	C11—N12—C14—C16	−179.48 (16)
N5—N4—N3—C1	−178.25 (12)	C11—N13—C17—C18	−175.99 (19)
N5—N4—N3—C7	0.47 (15)	C12—C13—C14—C16	178.44 (16)
N5—C8—C9—C9A	−117.03 (18)	C14—C13—C12—C15	−176.45 (17)
N11—C11—N12—C14	0.4 (2)	C17—C18—C19—C19A	−13.1 (2)
N11—C11—N13—N14	−10.54 (17)	C18—C19—C19A—C19B	−178.96 (16)

### Di-µ-iodido-bis{[4,6-dimethyl-2-(4-propyl-1H-1,2,3-triazol-1-yl-κN2)pyrimidine-κN1]copper(I)} (II) Hydrogen-bond geometry (Å, º)

**Table:**

*D*—H···*A*	*D*—H	H···*A*	*D*···*A*	*D*—H···*A*
C15—H15C···I2^i^	1.06 (2)	3.02 (2)	4.0596 (19)	165 (2)
C16—H16C···N5^i^	1.03 (3)	2.54 (3)	3.492 (2)	155 (3)
C17—H17···I1^ii^	1.09 (2)	2.82 (2)	3.9016 (16)	171 (2)

Symmetry codes: (i) −*x*+1, −*y*+1, −*z*; (ii) −*x*+1/2, *y*−1/2, −*z*+1/2.

### Bis[4,6-dimethyl-2-(4-propyl-1H-1,2,3-triazol-1-yl-κN2)pyrimidine-κN1](nitrato-κO)silver(I) (III) Crystal data

**Table:**

[Ag(NO_3_)(C_11_H_15_N_5_)_2_]	*Z* = 2
*M**_r_* = 604.42	*F*(000) = 622.613
Triclinic, *P*1	*D*_x_ = 1.568 Mg m^−^^3^
*a* = 10.4356 (2) Å	Cu *K*α radiation, λ = 1.54184 Å
*b* = 11.4639 (2) Å	Cell parameters from 19021 reflections
*c* = 11.8944 (2) Å	θ = 3.8–74.8°
α = 97.213 (1)°	µ = 6.72 mm^−^^1^
β = 100.878 (2)°	*T* = 100 K
γ = 110.519 (2)°	Plate, colourless
*V* = 1279.99 (5) Å^3^	0.35 × 0.1 × 0.06 × 0.07 (radius) mm

### Bis[4,6-dimethyl-2-(4-propyl-1H-1,2,3-triazol-1-yl-κN2)pyrimidine-κN1](nitrato-κO)silver(I) (III) Data collection

**Table:**

SuperNova, Dual, Cu at home/near, Pilatus 200K diffractometer	5150 independent reflections
Radiation source: micro-focus sealed X-ray tube, SuperNova (Cu) X-ray Source	4952 reflections with *I*≥ 2u(*I*)
Mirror monochromator	*R*_int_ = 0.045
Detector resolution: 5.8140 pixels mm^-1^	θ_max_ = 74.9°, θ_min_ = 3.9°
ω scans	*h* = −12→13
Absorption correction: for a sphere (CrysAlisPro; Rigaku OD, 2024)	*k* = −14→14
*T*_min_ = 0.369, *T*_max_ = 0.429	*l* = −12→14
24918 measured reflections	

### Bis[4,6-dimethyl-2-(4-propyl-1H-1,2,3-triazol-1-yl-κN2)pyrimidine-κN1](nitrato-κO)silver(I) (III) Refinement

**Table:**

Refinement on *F*^2^	0 constraints
Least-squares matrix: full	Primary atom site location: dual
*R*[*F*^2^ > 2σ(*F*^2^)] = 0.019	All H-atom parameters refined
*wR*(*F*^2^) = 0.046	*w* = 1/[σ^2^(*F*_o_^2^) + (0.0193*P*)^2^ + 0.0697*P*] where *P* = (*F*_o_^2^ + 2*F*_c_^2^)/3
*S* = 1.09	(Δ/σ)_max_ = 0.001
5150 reflections	Δρ_max_ = 0.44 e Å^−^^3^
604 parameters	Δρ_min_ = −0.53 e Å^−^^3^
213 restraints	

### Bis[4,6-dimethyl-2-(4-propyl-1H-1,2,3-triazol-1-yl-κN2)pyrimidine-κN1](nitrato-κO)silver(I) (III) Fractional atomic coordinates and isotropic or equivalent isotropic displacement parameters (Å^2^)

**Table:**

	*x*	*y*	*z*	*U*_iso_*/*U*_eq_	
Ag1	0.820169 (11)	0.274095 (9)	0.652795 (9)	0.01919 (4)	
O1	0.98453 (12)	0.28099 (10)	0.82732 (10)	0.0247 (2)	
O2	1.07648 (14)	0.19801 (11)	0.70706 (10)	0.0311 (3)	
O3	1.14152 (15)	0.20850 (14)	0.89257 (11)	0.0435 (4)	
N1	0.84264 (12)	0.49733 (11)	0.70430 (10)	0.0159 (2)	
N2	0.86194 (13)	0.68586 (11)	0.63032 (11)	0.0168 (2)	
N3	0.83810 (13)	0.50155 (10)	0.50839 (11)	0.0151 (2)	
N4	0.81674 (13)	0.37639 (11)	0.48612 (11)	0.0178 (3)	
N5	0.79681 (13)	0.34071 (11)	0.37402 (11)	0.0187 (3)	
N6	1.06852 (14)	0.22885 (11)	0.80911 (11)	0.0199 (3)	
N11	0.71355 (13)	0.05006 (11)	0.56247 (11)	0.0158 (2)	
N12	0.54773 (13)	−0.15346 (11)	0.57021 (11)	0.0177 (3)	
N13	0.56326 (13)	0.02915 (10)	0.68824 (11)	0.0160 (2)	
N14	0.61848 (13)	0.15592 (11)	0.73109 (11)	0.0187 (3)	
N15	0.55407 (13)	0.17826 (11)	0.81030 (11)	0.0196 (3)	
C1	0.85017 (14)	0.56597 (12)	0.62239 (12)	0.0141 (3)	
C2	0.84648 (15)	0.55718 (13)	0.81086 (13)	0.0172 (3)	
C3	0.86049 (16)	0.68385 (14)	0.83031 (14)	0.0184 (3)	
H3	0.866 (2)	0.7323 (16)	0.9166 (17)	0.031 (5)	
C4	0.86842 (15)	0.74657 (13)	0.73711 (13)	0.0167 (3)	
C5	0.8299 (2)	0.48058 (16)	0.90333 (14)	0.0228 (3)	
H5a	0.723 (3)	0.413 (2)	0.884 (2)	0.051 (6)	
H5b	0.904 (2)	0.431 (2)	0.9082 (19)	0.045 (6)	
H5c	0.851 (2)	0.5402 (18)	0.9874 (18)	0.041 (5)	
C6	0.88167 (19)	0.88208 (15)	0.75122 (15)	0.0233 (3)	
H6a	0.983 (3)	0.946 (2)	0.804 (2)	0.061 (7)	
H6b	0.872 (2)	0.9137 (18)	0.6648 (18)	0.042 (6)	
H6c	0.801 (3)	0.895 (2)	0.789 (2)	0.060 (7)	
C7	0.83270 (15)	0.54581 (13)	0.40746 (13)	0.0167 (3)	
H7	0.852 (2)	0.6435 (16)	0.4036 (16)	0.028 (5)	
C8	0.80563 (16)	0.44186 (13)	0.32139 (13)	0.0177 (3)	
C9	0.78100 (18)	0.42736 (15)	0.19221 (13)	0.0217 (3)	
H9a	0.837 (2)	0.5186 (15)	0.1701 (18)	0.046 (6)	
H9b	0.827 (2)	0.3602 (17)	0.1628 (17)	0.041 (5)	
C9A	0.62388 (19)	0.37629 (17)	0.12867 (15)	0.0302 (4)	
H9Aa	0.612 (2)	0.3528 (19)	0.0334 (13)	0.044 (5)	
H9Ab	0.563 (2)	0.2875 (17)	0.1564 (19)	0.056 (6)	
C9B	0.5555 (3)	0.4708 (3)	0.1527 (2)	0.0625 (8)	
H9Ba	0.4427 (19)	0.431 (3)	0.105 (2)	0.082 (9)	
H9Bb	0.558 (3)	0.488 (3)	0.2457 (16)	0.095 (9)	
H9Bc	0.615 (4)	0.564 (2)	0.136 (4)	0.137 (16)	
C11	0.61194 (15)	−0.02750 (13)	0.60129 (12)	0.0157 (3)	
C12	0.75769 (15)	−0.00653 (13)	0.47944 (13)	0.0162 (3)	
C13	0.69638 (16)	−0.13803 (13)	0.43880 (13)	0.0182 (3)	
H13	0.7350 (19)	−0.1794 (16)	0.3740 (17)	0.031 (5)	
C14	0.59069 (15)	−0.20954 (13)	0.48705 (13)	0.0181 (3)	
C15	0.87396 (17)	0.07668 (15)	0.43483 (15)	0.0205 (3)	
H15a	0.849 (3)	0.152 (2)	0.402 (2)	0.052 (6)	
H15b	0.971 (2)	0.118 (2)	0.505 (2)	0.048 (6)	
H15c	0.891 (2)	0.0202 (19)	0.364 (2)	0.051 (6)	
C16	0.52013 (19)	−0.35095 (15)	0.44909 (17)	0.0256 (4)	
H16a	0.467 (3)	−0.390 (2)	0.513 (2)	0.069 (7)	
H16b	0.440 (3)	−0.375 (2)	0.366 (3)	0.070 (8)	
H16c	0.591 (2)	−0.396 (2)	0.438 (2)	0.055 (6)	
C17	0.46070 (16)	−0.03085 (14)	0.74148 (13)	0.0167 (3)	
H17	0.405 (2)	−0.1290 (18)	0.7159 (17)	0.036 (5)	
C18	0.45573 (15)	0.06547 (13)	0.81952 (13)	0.0171 (3)	
C19	0.36502 (17)	0.06054 (14)	0.90344 (15)	0.0213 (3)	
H19a	0.4303 (19)	0.1121 (17)	0.9909 (13)	0.036 (5)	
H19b	0.297 (2)	0.1103 (19)	0.8763 (19)	0.054 (6)	
C19A	0.27472 (17)	−0.07423 (15)	0.91147 (14)	0.0218 (3)	
H19c	0.1998 (18)	−0.1238 (18)	0.8248 (13)	0.037 (5)	
H19d	0.341 (2)	−0.1296 (18)	0.9260 (18)	0.043 (5)	
C19B	0.19656 (19)	−0.07269 (18)	1.00689 (15)	0.0269 (4)	
H19e	0.129 (2)	−0.0188 (19)	0.995 (2)	0.052 (6)	
H19f	0.130 (2)	−0.1665 (16)	1.014 (2)	0.054 (6)	
H19g	0.270 (2)	−0.0221 (19)	1.0907 (15)	0.048 (6)	

### Bis[4,6-dimethyl-2-(4-propyl-1H-1,2,3-triazol-1-yl-κN2)pyrimidine-κN1](nitrato-κO)silver(I) (III) Atomic displacement parameters (Å^2^)

**Table:**

	*U*^11^	*U*^22^	*U*^33^	*U*^12^	*U*^13^	*U*^23^
Ag1	0.02353 (7)	0.01442 (6)	0.01953 (7)	0.00672 (4)	0.00509 (4)	0.00533 (4)
O1	0.0272 (6)	0.0258 (5)	0.0242 (6)	0.0152 (5)	0.0048 (5)	0.0037 (5)
O2	0.0439 (7)	0.0323 (6)	0.0199 (6)	0.0162 (5)	0.0126 (5)	0.0038 (5)
O3	0.0589 (9)	0.0695 (9)	0.0221 (7)	0.0510 (8)	0.0050 (6)	0.0093 (6)
N1	0.0206 (6)	0.0153 (6)	0.0117 (6)	0.0068 (5)	0.0044 (5)	0.0028 (5)
N2	0.0240 (6)	0.0141 (5)	0.0133 (6)	0.0080 (5)	0.0052 (5)	0.0035 (5)
N3	0.0205 (6)	0.0130 (5)	0.0119 (6)	0.0060 (5)	0.0049 (5)	0.0025 (5)
N4	0.0260 (7)	0.0133 (5)	0.0139 (6)	0.0077 (5)	0.0045 (5)	0.0026 (5)
N5	0.0269 (7)	0.0139 (6)	0.0141 (6)	0.0071 (5)	0.0048 (5)	0.0009 (5)
N6	0.0268 (7)	0.0197 (6)	0.0163 (7)	0.0126 (5)	0.0049 (5)	0.0039 (5)
N11	0.0187 (6)	0.0143 (5)	0.0164 (6)	0.0072 (5)	0.0064 (5)	0.0044 (5)
N12	0.0199 (6)	0.0142 (5)	0.0194 (6)	0.0070 (5)	0.0061 (5)	0.0025 (5)
N13	0.0192 (6)	0.0139 (5)	0.0154 (6)	0.0072 (5)	0.0045 (5)	0.0022 (5)
N14	0.0211 (6)	0.0153 (5)	0.0189 (6)	0.0060 (5)	0.0060 (5)	0.0021 (5)
N15	0.0230 (6)	0.0154 (6)	0.0204 (7)	0.0072 (5)	0.0079 (5)	0.0005 (5)
C1	0.0179 (7)	0.0137 (6)	0.0109 (7)	0.0061 (5)	0.0036 (5)	0.0030 (5)
C2	0.0222 (7)	0.0171 (7)	0.0117 (7)	0.0067 (6)	0.0046 (6)	0.0031 (6)
C3	0.0248 (8)	0.0169 (7)	0.0133 (8)	0.0083 (6)	0.0049 (6)	0.0012 (6)
H3	0.043 (11)	0.021 (9)	0.029 (10)	0.008 (6)	0.014 (7)	0.004 (7)
C4	0.0219 (7)	0.0161 (7)	0.0126 (7)	0.0086 (6)	0.0040 (6)	0.0013 (6)
C5	0.0330 (9)	0.0214 (8)	0.0134 (8)	0.0091 (7)	0.0065 (7)	0.0044 (6)
H5a	0.057 (13)	0.039 (11)	0.048 (12)	0.006 (7)	0.010 (8)	0.013 (7)
H5b	0.056 (12)	0.050 (11)	0.039 (12)	0.034 (8)	0.012 (7)	0.003 (7)
H5c	0.061 (13)	0.037 (10)	0.029 (11)	0.020 (7)	0.014 (7)	0.016 (7)
C6	0.0334 (9)	0.0186 (7)	0.0195 (9)	0.0128 (7)	0.0067 (7)	0.0016 (7)
H6a	0.075 (14)	0.044 (11)	0.056 (13)	0.026 (8)	0.000 (8)	0.001 (8)
H6b	0.068 (13)	0.036 (10)	0.027 (11)	0.034 (7)	−0.002 (7)	0.008 (7)
H6c	0.072 (14)	0.049 (11)	0.073 (14)	0.024 (8)	0.040 (8)	0.021 (8)
C7	0.0240 (8)	0.0132 (7)	0.0122 (7)	0.0061 (6)	0.0047 (6)	0.0030 (6)
H7	0.045 (11)	0.018 (9)	0.026 (10)	0.018 (6)	0.004 (7)	0.008 (6)
C8	0.0235 (7)	0.0153 (6)	0.0129 (7)	0.0058 (6)	0.0050 (6)	0.0021 (6)
C9	0.0297 (8)	0.0209 (7)	0.0135 (8)	0.0074 (6)	0.0078 (6)	0.0033 (6)
H9a	0.051 (12)	0.042 (11)	0.038 (11)	0.007 (7)	0.014 (7)	0.012 (7)
H9b	0.046 (11)	0.046 (10)	0.034 (11)	0.026 (7)	0.013 (7)	−0.005 (7)
C9A	0.0328 (10)	0.0377 (9)	0.0173 (8)	0.0121 (8)	0.0038 (7)	0.0036 (7)
H9Aa	0.053 (12)	0.060 (11)	0.018 (4)	0.024 (7)	0.006 (3)	0.003 (3)
H9Ab	0.065 (12)	0.049 (7)	0.034 (11)	−0.001 (4)	0.008 (6)	0.014 (4)
C9B	0.0716 (18)	0.099 (2)	0.0308 (12)	0.0636 (17)	−0.0050 (12)	−0.0042 (13)
H9Ba	0.062 (14)	0.114 (16)	0.065 (15)	0.036 (8)	0.002 (8)	0.014 (8)
H9Bb	0.104 (17)	0.126 (16)	0.069 (15)	0.065 (9)	0.017 (9)	0.013 (9)
H9Bc	0.13 (2)	0.13 (2)	0.15 (2)	0.046 (9)	0.037 (9)	0.032 (9)
C11	0.0172 (7)	0.0156 (6)	0.0148 (7)	0.0069 (5)	0.0042 (5)	0.0028 (6)
C12	0.0183 (7)	0.0163 (7)	0.0152 (7)	0.0071 (6)	0.0050 (6)	0.0045 (6)
C13	0.0212 (7)	0.0165 (7)	0.0178 (8)	0.0086 (6)	0.0053 (6)	0.0024 (6)
H13	0.023 (9)	0.028 (9)	0.032 (8)	0.003 (5)	0.011 (4)	−0.009 (4)
C14	0.0189 (7)	0.0154 (7)	0.0197 (8)	0.0071 (6)	0.0052 (6)	0.0013 (6)
C15	0.0245 (8)	0.0213 (7)	0.0185 (8)	0.0093 (6)	0.0097 (7)	0.0062 (7)
H15a	0.074 (14)	0.038 (10)	0.060 (13)	0.030 (7)	0.025 (8)	0.027 (7)
H15b	0.032 (11)	0.057 (12)	0.047 (12)	0.006 (7)	0.012 (7)	0.011 (8)
H15c	0.048 (12)	0.043 (11)	0.056 (13)	0.011 (7)	0.021 (8)	−0.002 (7)
C16	0.0262 (9)	0.0155 (7)	0.0341 (10)	0.0081 (6)	0.0092 (8)	−0.0006 (7)
H16a	0.087 (15)	0.044 (12)	0.074 (14)	0.016 (8)	0.037 (8)	0.008 (8)
H16b	0.083 (15)	0.034 (11)	0.078 (15)	0.016 (8)	0.003 (8)	0.001 (8)
H16c	0.035 (11)	0.043 (11)	0.086 (14)	0.017 (7)	0.018 (8)	0.004 (8)
C17	0.0193 (7)	0.0130 (7)	0.0172 (7)	0.0051 (6)	0.0063 (6)	0.0019 (6)
H17	0.032 (11)	0.030 (10)	0.032 (11)	0.002 (6)	0.002 (7)	0.001 (7)
C18	0.0181 (7)	0.0160 (6)	0.0176 (7)	0.0070 (5)	0.0054 (6)	0.0022 (6)
C19	0.0224 (8)	0.0211 (7)	0.0212 (8)	0.0086 (6)	0.0090 (6)	0.0016 (6)
H19a	0.043 (11)	0.042 (10)	0.014 (9)	0.014 (7)	0.004 (7)	−0.008 (7)
H19b	0.065 (13)	0.058 (11)	0.056 (13)	0.038 (8)	0.020 (8)	0.020 (8)
C19A	0.0236 (8)	0.0238 (7)	0.0172 (8)	0.0077 (6)	0.0072 (6)	0.0028 (6)
H19c	0.035 (8)	0.047 (10)	0.019 (5)	0.005 (5)	0.005 (3)	0.001 (3)
H19d	0.045 (8)	0.048 (8)	0.047 (12)	0.030 (4)	0.013 (6)	0.013 (6)
C19B	0.0248 (9)	0.0383 (10)	0.0176 (8)	0.0113 (7)	0.0069 (7)	0.0062 (7)
H19e	0.049 (12)	0.062 (12)	0.048 (12)	0.026 (7)	0.012 (8)	0.012 (8)
H19f	0.054 (13)	0.049 (11)	0.057 (13)	0.006 (7)	0.027 (8)	0.023 (8)
H19g	0.049 (12)	0.052 (11)	0.042 (12)	0.020 (7)	0.013 (7)	0.006 (7)

### Bis[4,6-dimethyl-2-(4-propyl-1H-1,2,3-triazol-1-yl-κN2)pyrimidine-κN1](nitrato-κO)silver(I) (III) Geometric parameters (Å, º)

**Table:**

Ag1—O1	2.4036 (11)	C6—H6c	1.07 (2)
Ag1—N1	2.4720 (11)	C7—H7	1.075 (16)
Ag1—N4	2.4276 (12)	C7—C8	1.377 (2)
Ag1—N11	2.4168 (11)	C8—C9	1.487 (2)
Ag1—N14	2.4881 (13)	C9—H9a	1.096 (14)
O1—N6	1.2564 (16)	C9—H9b	1.096 (13)
O2—N6	1.2475 (17)	C9—C9A	1.533 (2)
O3—N6	1.2319 (17)	C9A—H9Aa	1.104 (14)
N1—C1	1.3240 (18)	C9A—H9Ab	1.124 (14)
N1—C2	1.3499 (19)	C9A—C9B	1.518 (3)
N2—C1	1.3258 (17)	C9B—H9Ba	1.105 (16)
N2—C4	1.3472 (19)	C9B—H9Bb	1.093 (17)
N3—N4	1.3553 (15)	C9B—H9Bc	1.103 (17)
N3—C1	1.4208 (18)	C12—C13	1.3927 (19)
N3—C7	1.3599 (19)	C12—C15	1.491 (2)
N4—N5	1.2989 (18)	C13—H13	1.068 (17)
N5—C8	1.3690 (19)	C13—C14	1.393 (2)
N11—C11	1.3248 (19)	C14—C16	1.493 (2)
N11—C12	1.3524 (18)	C15—H15a	1.08 (2)
N12—C11	1.3286 (17)	C15—H15b	1.09 (2)
N12—C14	1.3424 (19)	C15—H15c	1.08 (2)
N13—N14	1.3491 (15)	C16—H16a	1.08 (3)
N13—C11	1.4178 (18)	C16—H16b	1.10 (3)
N13—C17	1.3670 (19)	C16—H16c	1.06 (2)
N14—N15	1.3054 (17)	C17—H17	1.041 (18)
N15—C18	1.3729 (19)	C17—C18	1.373 (2)
C2—C3	1.3920 (19)	C18—C19	1.493 (2)
C2—C5	1.489 (2)	C19—H19a	1.092 (13)
C3—H3	1.085 (19)	C19—H19b	1.083 (14)
C3—C4	1.395 (2)	C19—C19A	1.528 (2)
C4—C6	1.495 (2)	C19A—H19c	1.111 (14)
C5—H5a	1.07 (2)	C19A—H19d	1.093 (13)
C5—H5b	1.10 (2)	C19A—C19B	1.519 (2)
C5—H5c	1.08 (2)	C19B—H19e	1.091 (15)
C6—H6a	1.07 (2)	C19B—H19f	1.080 (14)
C6—H6b	1.13 (2)	C19B—H19g	1.084 (15)
			
N1—Ag1—O1	96.11 (4)	H9a—C9—C8	110.1 (11)
N4—Ag1—O1	139.22 (4)	H9b—C9—C8	106.7 (11)
N4—Ag1—N1	66.92 (4)	H9b—C9—H9a	108.0 (16)
N11—Ag1—O1	102.96 (4)	C9A—C9—C8	113.00 (14)
N11—Ag1—N1	158.63 (4)	C9A—C9—H9a	109.6 (12)
N11—Ag1—N4	103.02 (4)	C9A—C9—H9b	109.3 (11)
N14—Ag1—O1	90.42 (4)	H9Aa—C9A—C9	109.2 (11)
N14—Ag1—N1	103.00 (4)	H9Ab—C9A—C9	111.0 (13)
N14—Ag1—N4	128.58 (4)	H9Ab—C9A—H9Aa	108.2 (16)
N14—Ag1—N11	67.68 (4)	C9B—C9A—C9	112.91 (17)
N6—O1—Ag1	113.96 (9)	C9B—C9A—H9Aa	108.5 (11)
C1—N1—Ag1	119.00 (9)	C9B—C9A—H9Ab	106.8 (13)
C2—N1—Ag1	125.31 (9)	H9Ba—C9B—C9A	110.8 (16)
C2—N1—C1	115.68 (12)	H9Bb—C9B—C9A	109.3 (16)
C4—N2—C1	115.32 (12)	H9Bb—C9B—H9Ba	106 (2)
C1—N3—N4	121.07 (11)	H9Bc—C9B—C9A	112 (2)
C7—N3—N4	110.28 (11)	H9Bc—C9B—H9Ba	113 (3)
C7—N3—C1	128.40 (11)	H9Bc—C9B—H9Bb	105 (3)
N3—N4—Ag1	116.72 (9)	N12—C11—N11	128.89 (13)
N5—N4—Ag1	135.76 (9)	N13—C11—N11	116.93 (12)
N5—N4—N3	107.51 (11)	N13—C11—N12	114.18 (12)
C8—N5—N4	109.70 (11)	C13—C12—N11	120.47 (13)
O2—N6—O1	119.70 (13)	C15—C12—N11	117.57 (13)
O3—N6—O1	119.35 (13)	C15—C12—C13	121.95 (13)
O3—N6—O2	120.95 (13)	H13—C13—C12	118.3 (9)
C11—N11—Ag1	119.51 (9)	C14—C13—C12	118.47 (14)
C12—N11—Ag1	124.61 (9)	C14—C13—H13	123.2 (9)
C12—N11—C11	115.65 (12)	C13—C14—N12	121.05 (13)
C14—N12—C11	115.46 (12)	C16—C14—N12	117.02 (14)
C11—N13—N14	121.94 (12)	C16—C14—C13	121.93 (14)
C17—N13—N14	110.53 (11)	H15a—C15—C12	112.0 (12)
C17—N13—C11	127.52 (12)	H15b—C15—C12	109.3 (11)
N13—N14—Ag1	113.59 (9)	H15b—C15—H15a	109.1 (17)
N15—N14—Ag1	138.64 (9)	H15c—C15—C12	109.4 (11)
N15—N14—N13	107.50 (11)	H15c—C15—H15a	107.9 (18)
C18—N15—N14	109.40 (11)	H15c—C15—H15b	109.1 (17)
N2—C1—N1	129.13 (13)	H16a—C16—C14	109.9 (12)
N3—C1—N1	116.04 (11)	H16b—C16—C14	109.3 (12)
N3—C1—N2	114.78 (12)	H16b—C16—H16a	108 (2)
C3—C2—N1	120.45 (13)	H16c—C16—C14	113.1 (11)
C5—C2—N1	117.11 (13)	H16c—C16—H16a	107.9 (19)
C5—C2—C3	122.40 (14)	H16c—C16—H16b	108.9 (19)
H3—C3—C2	120.2 (10)	H17—C17—N13	119.9 (11)
C4—C3—C2	118.59 (14)	C18—C17—N13	104.55 (12)
C4—C3—H3	121.2 (10)	C18—C17—H17	135.6 (11)
C3—C4—N2	120.82 (13)	C17—C18—N15	108.02 (13)
C6—C4—N2	117.67 (13)	C19—C18—N15	121.80 (12)
C6—C4—C3	121.50 (13)	C19—C18—C17	130.18 (13)
H5a—C5—C2	109.6 (12)	H19a—C19—C18	110.2 (10)
H5b—C5—C2	109.9 (12)	H19b—C19—C18	107.7 (12)
H5b—C5—H5a	110.6 (17)	H19b—C19—H19a	106.8 (16)
H5c—C5—C2	111.0 (10)	C19A—C19—C18	114.12 (12)
H5c—C5—H5a	106.8 (17)	C19A—C19—H19a	108.3 (10)
H5c—C5—H5b	108.8 (16)	C19A—C19—H19b	109.6 (12)
H6a—C6—C4	111.7 (12)	H19c—C19A—C19	108.6 (10)
H6b—C6—C4	112.1 (10)	H19d—C19A—C19	110.0 (11)
H6b—C6—H6a	104.6 (17)	H19d—C19A—H19c	104.5 (15)
H6c—C6—C4	110.4 (12)	C19B—C19A—C19	111.48 (13)
H6c—C6—H6a	109.7 (19)	C19B—C19A—H19c	111.1 (10)
H6c—C6—H6b	108.1 (17)	C19B—C19A—H19d	110.9 (11)
H7—C7—N3	123.9 (10)	H19e—C19B—C19A	112.8 (12)
C8—C7—N3	104.78 (12)	H19f—C19B—C19A	113.4 (12)
C8—C7—H7	131.3 (10)	H19f—C19B—H19e	106.5 (17)
C7—C8—N5	107.72 (13)	H19g—C19B—C19A	110.6 (12)
C9—C8—N5	121.45 (13)	H19g—C19B—H19e	103.0 (16)
C9—C8—C7	130.76 (14)	H19g—C19B—H19f	109.9 (17)
			
Ag1—O1—N6—O2	11.37 (10)	N3—C7—C8—N5	−0.33 (13)
Ag1—O1—N6—O3	−168.35 (11)	N3—C7—C8—C9	176.69 (11)
Ag1—N1—C1—N2	179.77 (9)	N4—N5—C8—C7	−0.01 (13)
Ag1—N1—C1—N3	2.72 (10)	N4—N5—C8—C9	−177.37 (12)
Ag1—N1—C2—C3	179.53 (11)	N5—C8—C9—C9A	82.47 (16)
Ag1—N1—C2—C5	−2.63 (12)	N11—C11—N12—C14	−0.87 (18)
Ag1—N4—N3—C1	−5.00 (11)	N11—C11—N13—N14	−0.84 (15)
Ag1—N4—N3—C7	−179.70 (10)	N11—C11—N13—C17	−179.72 (12)
Ag1—N4—N5—C8	179.23 (14)	N11—C12—C13—C14	−1.06 (16)
Ag1—N11—C11—N12	−174.43 (9)	N12—C11—N13—N14	179.01 (11)
Ag1—N11—C11—N13	5.39 (10)	N12—C11—N13—C17	0.13 (16)
Ag1—N11—C12—C13	175.13 (11)	N12—C14—C13—C12	0.48 (17)
Ag1—N11—C12—C15	−4.34 (12)	N13—N14—N15—C18	−0.07 (13)
Ag1—N14—N13—C11	−3.80 (11)	N13—C17—C18—N15	0.06 (13)
Ag1—N14—N13—C17	175.25 (9)	N13—C17—C18—C19	−179.92 (11)
Ag1—N14—N15—C18	−173.33 (15)	N14—N15—C18—C17	0.00 (13)
N1—C1—N2—C4	0.67 (18)	N14—N15—C18—C19	179.99 (12)
N1—C1—N3—N4	1.54 (15)	N15—C18—C19—C19A	−173.13 (15)
N1—C1—N3—C7	175.20 (12)	C2—C3—C4—C6	179.21 (14)
N1—C2—C3—C4	0.88 (16)	C7—C8—C9—C9A	−94.20 (19)
N2—C1—N3—N4	−175.94 (12)	C8—C9—C9A—C9B	68.52 (18)
N2—C1—N3—C7	−2.28 (17)	C12—C13—C14—C16	−179.60 (14)
N2—C4—C3—C2	0.35 (16)	C17—C18—C19—C19A	6.85 (19)
N3—N4—N5—C8	0.36 (13)	C18—C19—C19A—C19B	174.20 (14)

### Bis[4,6-dimethyl-2-(4-propyl-1H-1,2,3-triazol-1-yl-κN2)pyrimidine-κN1](nitrato-κO)silver(I) (III) Hydrogen-bond geometry (Å, º)

**Table:**

*D*—H···*A*	*D*—H	H···*A*	*D*···*A*	*D*—H···*A*
C5—H5A···N15	1.07 (3)	2.57 (2)	3.516 (2)	146 (2)
C5—H5B···O1	1.11 (2)	2.34 (2)	3.358 (2)	152 (2)
C15—H15A···N5	1.08 (2)	2.46 (3)	3.517 (2)	167 (2)
C15—H15B···O2	1.09 (2)	2.36 (2)	3.333 (2)	148 (2)
C3—H3···O3^i^	1.09 (2)	2.31 (2)	3.378 (2)	167.6 (14)
C7—H7···O2^ii^	1.075 (18)	2.369 (18)	3.3044 (19)	144.6 (14)
C9—H9A···O1^ii^	1.10 (2)	2.39 (2)	3.449 (2)	162 (2)
C13—H13···O2^iii^	1.07 (2)	2.41 (2)	3.391 (2)	152.2 (15)
C17—H17···N5^iv^	1.04 (2)	2.53 (2)	3.524 (2)	160.0 (17)
C19—H19B···O3^v^	1.09 (2)	2.30 (2)	3.324 (2)	157 (2)

Symmetry codes: (i) −*x*+2, −*y*+1, −*z*+2; (ii) −*x*+2, −*y*+1, −*z*+1; (iii) −*x*+2, −*y*, −*z*+1; (iv) −*x*+1, −*y*, −*z*+1; (v) *x*−1, *y*, *z*.
